# Investigation of Host-Guest Inclusion Complexes Between Carmustine and α-Cyclodextrin: Synthesis, Characterization, and Evaluation

**DOI:** 10.3390/ijms26199386

**Published:** 2025-09-25

**Authors:** Katarzyna Strzelecka, Dominika Janiec, Jan Sobieraj, Adam Kasiński, Marzena Kuras, Aldona Zalewska, Łukasz Szeleszczuk, Marcin Sobczak, Marta K. Dudek, Ewa Oledzka

**Affiliations:** 1 Department of Pharmaceutical Chemistry and Biomaterials, Faculty of Pharmacy, Medical University of Warsaw, 1 Banacha Street, 02-097 Warsaw, Poland; 2 Doctoral School, Medical University of Warsaw, 81 Żwirki i Wigury Street, 02-093 Warsaw, Poland; 3 Faculty of Chemistry, Warsaw University of Technology, 3 Noakowskiego Street, 00-664 Warsaw, Poland; 4 Department of Organic and Physical Chemistry, Faculty of Pharmacy, Medical University of Warsaw, 1 Banacha Street, 02-097 Warsaw, Poland; lukasz.szeleszczuk@wum.edu.pl; 5 Structural Studies Department, Centre of Molecular and Macromolecular Studies, Polish Academy of Sciences, 112 Sienkiewicza Street, 90-363 Łódź, Poland

**Keywords:** carmustine, α-cyclodextrin, solid state inclusion complex, drug delivery systems, mechanochemical synthesis

## Abstract

Carmustine (BCNU) is a powerful alkylating agent primarily used in the chemotherapeutic treatment of malignant brain tumors. However, its clinical application faces significant constraints due to its lipophilicity, low thermal stability, and rapid degradation in physiological environments. To tackle these challenges, our research aimed at the development and detailed characterization of α-cyclodextrin (α-CD) inclusion complexes (ICs) with BCNU employing three different synthesis techniques: co-grinding, cryomilling, and co-precipitation. The selected synthetic methods displayed variations dependent on the technique used, affecting the efficiency, inclusion ratios, and drug-loading capacities, with co-precipitation achieving the most favorable complexation parameters. Structural elucidation through ^1^H NMR chemical shifts analysis indicated that only partial inclusion of BCNU occurred within α-CD in ICs produced via co-grinding, while cryomilling and co-precipitation allowed for complete inclusion. Multimodal spectroscopic analyses (FT-IR, UV-Vis, ^13^C CP MAS NMR, and ESI-MS) further substantiated the effective encapsulation of BCNU within α-CD, and systematic solubility assessments via Job’s continuous variation and the Benesi-Hildebrand method revealed a 1:1 host-guest stoichiometry. The ICs obtained were evaluated for BCNU release in vitro at pH levels of 4, 5, 6.5, and 7.4. The mechanism of BCNU drug release was determined to be Fickian diffusion, with the highest cumulative release noted in the acidic microenvironment. These findings collectively validate the effectiveness of α-CD as a functional excipient for the modulation of BCNU’s physicochemical properties through non-covalent complexation. This strategy shows potential for increasing the stability and solubility of BCNU, which may enhance its therapeutic effectiveness in the treatment of brain tumors.

## 1. Introduction

Over the past years, significant advancements have been made in the field of medicine and cancer therapeutics. Nonetheless, cancer continues to pose a formidable health challenge owing to its intricate characteristics [1]. The American Cancer Society projects that the incidence of cancer cases will escalate to 35 million worldwide by the year 2050 [2]. Primary brain cancers are among the most concerning malignancies, which are especially difficult to treat, yielding an overall five-year survival ratio of merely 35% [3]. Glioblastoma multiforme (GBM) is a brain neoplasm predominantly viewed as unmanageable and has been the research topic for almost a hundred years. In present clinical practice, the conventional therapeutic methods continue to encompass surgical resection, chemotherapy and radiation therapy. Nonetheless, the inherent tendencies for invasion and metastasis, coupled with significant resistance to chemotherapeutic agents and difficulties with performing surgical removal, contribute to a persistently unsatisfactory survival rate among patients with GBM [4]. GBM accounts for about 45.2% of all central nervous system tumors, and 54.4% of all malignant gliomas, highlighting an urgent demand for effective treatment alternatives [5].

A nanotechnology-based strategy seems to be a highly suitable solution to tackle the challenges associated with this type of brain tumor. This multidisciplinary field utilizes advanced materials and systems with improved characteristics and functionalities, enabling various transformative applications [6]. As a specialized area within nanotechnology, nanopharmacology focuses on developing of nanocarriers designed to optimize drug delivery and enhance therapeutic efficacy. By employing nanostructures, researchers aim to improve the bioavailability of active substances, reduce side effects, and enable targeted delivery to specific cellular sites. This targeted approach is particularly important in the treatment of complex diseases, such as cancer, where minimizing collateral damage to healthy tissues can significantly improve patient outcomes [7]. The potential of nanopharmacology is being rapidly explored, as research in this area has surged over the years. Various research efforts are exploring the use of nanomaterials in drug formulations, imaging, and diagnostics, with many promising candidates making their way into clinical trials and even reaching the market. This innovative field not only holds the promise of revolutionizing traditional therapeutic strategies, but also paves the way for personalized medicine, ultimately leading to more effective treatments tailored to the individual patient’s needs [8].

The therapeutic agents currently administered for GBM consist of temozolomide (TMZ), bevacizumab, and carmustine (BCNU, bis-chloronitrosourea). Notably, BCNU—an alkylating chemotherapeutic agent, requires particular attention. BCNU is a broad-spectrum alkylating agent that induces crosslinking in the DNA and RNA strands, which prevents cells from dividing and consequently leads to their death. Additionally, it attaches to and alters glutathione reductase, resulting in the death of tumor cells [9]. The pharmacological activity of BCNU is strongly connected to its physicochemical properties. Due to the low molecular weight, high lipophilicity and lack of ionization at physiological pH, BCNU excellently crosses the blood–brain barrier (BBB) [10,11]. However, the bioavailability of BCNU leaves much to desire, as just after 15 min post intravenous administration, it undergoes decomposition, resulting in a negligible concentration in the blood [10]. Considering the low molecular weight of the BCNU molecule, its ability to permeate and penetrate both tumor and healthy cells is enhanced. Additionally, its rapid degradation may necessitate higher dosages, potentially leading to significant side effects. These include pulmonary toxicity, leukopenia, thrombocytopenia, ocular toxicity and bone marrow suppression [9,11]. Furthermore, when analyzing the physicochemical properties of BCNU, it is essential to highlight its low thermal stability, indicated by a melting temperature (*T*_m_) of 31 °C, along with its stability in solutions that varies with pH. These aspects considerably limit its potential for clinical use [12,13]. As it was described by Montgomery et al., the thermal decomposition of molten BCNU at 50 °C with a restricted quantity of water leads to the formation of 2-chloroethanol and 1,3-bis(2-chloroethyl)urea, whereas reactions occurring in aqueous solutions resulted in an extraordinary decomposition that yielded acetaldehyde, HCl, N_2_ and 2-chloroethyl isocyanate derivatives [14]. Moreover, the study conducted by Laskar and Ayres on the degradation of BCNU in aqueous media indicated that a minimal degradation rate is observed in the pH value range between 5.2 and 5.5 [15].

In light of the limitations posed by BCNU, the researchers directed their efforts towards innovative strategies for the efficient administration of this antineoplastic agent. The beneficial method for the direct delivery of BCNU which has been developed by Eisai Inc. and approved by the U.S. Food and Drug Administration (FDA) in 1996, involves the utilization of drug-loaded polymeric wafers. Gliadel^®^ is a biodegradable polymeric implant for intracranial use, composed of poly[1,3-bis(*p*-carboxyphenoxy)propane sebacic acid], that has been employed in the treatment of GBM [16]. Gliadel^®^ has been proven to significantly enhance survival prospects in patients both with newly diagnosed high-grade gliomas and those with recurrent malignancies [17,18]. Nonetheless, the studies have demonstrated that Gliadel^®^ wafers may suffer from the phenomenon known as the “sink effect”. This occurs when the active substance is diminished in the systemic circulation due to excessive diffusion, wherein the concentration of the drug precipitously decreases, subsequently to its release from the wafer [18]. Furthermore, the opinions regarding the usage of Gliadel^®^ are not consistent throughout the medical professionals. As the Gliadel^®^ may not adequately adapt to the postoperative anatomical cavity, its inaccurate placement may lead to severe complications that include cerebral oedema, cerebrospinal fluid leakage, intercranial hypertension, and epileptic seizures [19]. Furthermore, the wafers may cause an allergic reaction and inflammation, potentially leading to irreversible hydrocephalus, a condition that has been recently highlighted by Kawaguchi et al. [20]. Additionally, the limited capacity of the resection cavity may restrain the quantity of Gliadel^®^ that can be utilized, thereby hindering the attainment of an effective drug concentration. Consequently, the role of Gliadel^®^ in the clinical practice has been declining in importance throughout the years [19].

Consequently, the scientific community has focused on developing novel drug delivery systems (DDSs) for BCNU [21]. Until now, nanoformulations such as liposomes [11,22,23], polymeric nanoparticles [24,25] and hydrogels [26] have been reported. Significantly, the group of cyclic oligosaccharides known as cyclodextrins (CDs) has demonstrated considerable benefits in addressing the challenges posed by the adverse physicochemical properties of pharmaceutical products. The capacity of CDs to create inclusion complexes (ICs) has ushered in a plethora of applications within the pharmaceutical sector. The process of complexation with CDs can be employed to augment various properties of numerous pharmaceuticals, including improvement in stability, partition coefficient, biological efficacy, bioavailability, dissolution rate, volatility, and solubility [27,28]. CDs comprise α-D-glucopyranose units interconnected by a α-1,4 glycosidic bonds. The distinctive shape of a truncated cone, featuring a central cavity, is bestowed upon CD molecules by the chair conformation of the glucopyranose units. The exterior of CD features hydroxyl groups that provide hydrophilicity, while the interior is relatively hydrophobic, which makes CDs capable of solubilizing various compounds [29]. Thorough characterization of CD ICs, both in solution and solid forms, is a complex process requiring multiple analytical techniques. The results of these findings must be integrated and assessed collectively [30,31,32].

CD host-guest complexes are inherently flexible structures, which explains their polycrystallinity. This is why multiple molecular modeling investigations of such complexes employ molecular dynamics (MD) simulations at the molecular mechanics (MM) level. Nevertheless, the types of intermolecular forces that stabilize CD complexes, including hydrogen bonding, van der Waals forces, hydrophobic contacts, and dipole–dipole interactions, are more accurately characterized at the quantum chemical (QC) level. This is why over the past two decades, the quantity of research focused on the calculations of CD complexes using QC-based methods has consistently increased.

The endeavor to acquire and characterize ICs of BCNU with CDs holds significant implications for both scientific research and clinical applications. The complexation of BCNU with CDs could enhance the solubility of the drug, potentially leading to improved bioavailability. Furthermore, CDs may offer protection to BCNU against both chemical and photochemical degradation, thereby enhancing the drug’s stability. This complexation process might also mitigate the toxicity associated with BCNU, which is vital for ensuring patient safety. Additionally, encapsulating BCNU within the cavity of CDs could facilitate controlled drug release, thereby enhancing therapeutic efficacy while minimizing side effects. Collectively, these benefits could profoundly influence the effectiveness and safety profile of BCNU in the context of GBM therapy.

To our current knowledge, the synthesis and in-depth characterization of α-cyclodextrin (α-CD) ICs with BCNU have not been previously documented in scientific literature. Therefore, this paper introduces the first comprehensive experimental characterization of α-CD as a carrier for BCNU in the solid state. BCNU-α-CD ICs were prepared using three different synthesis methods and subsequently characterized using a range of analytical techniques, including proton nuclear magnetic resonance (^1^H NMR), Fourier-transform infrared spectroscopy (FTIR), ultraviolet-visible spectroscopy (UV-Vis), Electrospray Ionization Mass Spectrometry (ESI-MS), and Carbon-13 Cross-Polarization Magic Angle Spinning Nuclear Magnetic Resonance (^13^C CP MAS NMR), along with thermal analysis via differential scanning calorimetry (DSC). We assessed the inclusion ratio, drug loading (*DL*), stoichiometry, and the dissociation and formation coefficients of the resulting complexes. This study also examined how α-CD complexation affects the solubility of BCNU and the in vitro release profile of the drug in various pH settings. Moreover, in our investigation we employed MD simulations and quantum chemical (QC) density functional theory (DFT) calculations. Initial coordinates for the structures were obtained through MD, followed by optimization and analysis.

## 2. Results and Discussion

### 2.1. Synthesis of BCNU-α-CD ICs

The primary objective during the first stage of this investigation was to enhance the methodology for creating solid-state ICs of BCNU with α-CD.

Solid BCNU-α-CD ICs were prepared using three different synthesis methods. This step was executed to refine the complexation process, with the goal of optimizing product yield, and to obtain complexes characterized by the optimal inclusion ratios and *DL* values. The preparation of ICs in the solid state enabled the formation of stable, isolable materials, whose key properties can be directly evaluated in subsequent investigations.

The first method (C1) involved the co-grinding of an equimolar combination of α-CD and BCNU for 45 min in a porcelain mortar with a pestle, utilizing a few drops of a 1:1 mixture of water and ethanol. According to Jug and Mura, 45 min of co-grinding is a sufficient time to obtain CD ICs in many cases [33]. The second BCNU-α-CD IC was prepared by cryomilling equimolar mixture (C2). Due to the fact that BCNU exhibits low thermal stability and undergoes thermal decomposition at the temperature of 31 °C, the traditional milling or ball milling technique was inappropriate [34]. To avert the thermal decomposition of BCNU, the cryomilling process has been modified accordingly. Last but not least, co-precipitation method has been applied (C3). In this method, the BCNU ethanolic solution was added dropwise to the saturated solution of α-CD, what was followed by 4 h of mixing and precipitation at 4 °C. This method was also carried out using equimolar ratios of α-CD and BCNU.

### 2.2. Characterization of BCNU-α-CD ICs

The obtained complexes were characterized with the use of HPLC technique to determine the inclusion ratio and drug loading (*DL*). The collected data is presented in Table 1. It was found that the yield, inclusion ratio and *DL* strongly depend on the synthesis method, resulting in the highest yield for C1 IC, and the highest inclusion ratio and *DL* for C3 resulted products.

The comparative analysis of chosen synthesis techniques reveals that mechanochemical methods such as co-grinding, cryomilling and co-precipitation are all highly effective in terms of inclusion efficiency, drug loading capacity, and the process yield, which is consistent with existing literature [35]. The study conducted by Pereva et al. also focused on the comparison of different synthesis methods in preparation of ibuprofen-β-CD complexes in the solid state. The researchers used kneading, solid dispersion technique (which resembles the co-precipitation method) and ball milling. The authors elucidated that while all three methods were capable of producing ICs, the solid dispersion technique and solvent-assisted ball milling were markedly superior in achieving high yields and inclusion ratios. The yields of complexation of the mentioned methods were equal to 100% and 96.8%, respectively. Our results are consistent with the observations of Pereva et al. with the methods utilizing even small amounts of solvents being the most prominent [36].

#### 2.2.1. DSC Analysis

**Figure 1:**
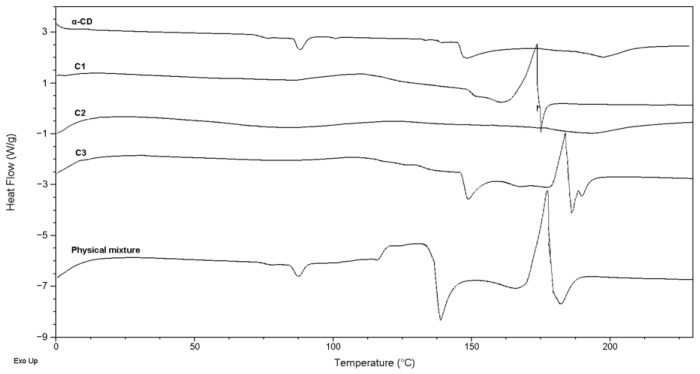
DSC thermograms of α-CD, C1, C2, C3 BCNU-α-CD ICs and physical mixture.

The thermal characteristics of BCNU, α-CD, and the resulting BCNU-α-CD ICs were examined using DSC, as illustrated in Figure 1 and Figure S1. Additionally, the physical mixture of the active compound with α-CD underwent the same analytical procedure for comparative purposes. The DSC thermogram revealed that the *T*_m_ of BCNU was 31.4 °C. In contrast, the thermogram for crystalline α-CD exhibited three distinct endothermic peaks at temperatures reaching a maximum of 85.5 °C, 148.1 °C, and 197.4 °C. These peaks correspond to the evaporation of water from the surface, within the interstices, and from the cavities, respectively [37,38]. Notably, distinctions in the endothermic peaks were identified between the complexed and uncomplexed α-CD. Analysis of the thermogram for the physical mixture of BCNU and α-CD indicated the presence of three separate endothermic peaks, with maximum temperatures recorded at 85.1 °C, 141.1 °C, and 182.2 °C. The third peak demonstrated an endothermic effect of 14.74 J/g and was slightly displaced from the peak observed in free α-CD. These peaks are representative of the α-CD component within the mixture. The findings indicate that an interaction between the two substrates has occurred; however, it does not support the occurrence of complexation [39,40].

The thermograms of BCNU-α-CD ICs exhibit notable differences that can be linked to their respective preparation techniques. For example, the C2 complex, which was synthesized using a cryogenic method, lacks any identifiable endothermic peaks. A closer analysis of this thermogram reveals two broad peaks at maximum temperatures of 82.4 °C and 194.0 °C, which are similar to those found in pure α-CD, indicating a lack of effective complex formation between the drug and the cyclodextrin. Conversely, the complex obtained through mortar grinding shows a slight peak at a maximum of 87.9 °C and a more significant peak at 173.6 °C, along with an endothermic effect measured at 10.04 J/g. Notably, the thermogram of the BCNU-α-CD IC derived from the co-precipitation method is the most promising, showcasing three endothermic peaks at a maximum of 148.5 °C, 184.9 °C (with an endothermic effect of 23.4 J/g), and a further peak at 189.6 °C. The observed shift in the peak associated with free α-CD, originally at 197.4 °C, to a lower temperature indicates that the disordering of the lattice has occurred, which might be caused by the BCNU-α-CD IC formation [41]. These results suggested that some of the water molecules bound to α-CD were replaced by BCNU molecules [42,43,44]. The successful incorporation of BCNU into the cavity of α-CD is noteworthy. It is essential to emphasize that the complexes formed did not display a peak indicative of the drug’s *T*_m_. Regrettably, this peak was similarly absent in the thermogram of the physical mixture. Consequently, we opted to validate the formation of the BCNU-α-CD ICs through alternative analytical techniques. In spite of this, the observed changes in thermal effects within the 160–180 °C range for samples C1, C3, and the physical mixture, which relate to the progressive dehydration of α-CD, together with the shifts in enthalpy, may imply the successful formation of ICs. Moreover, the absence of BCNU’s melting peak in the ICs samples acts as compelling proof of the drug’s incorporation into the cavity of α-CD.

#### 2.2.2. ^1^H NMR Analysis

**Figure 2:**
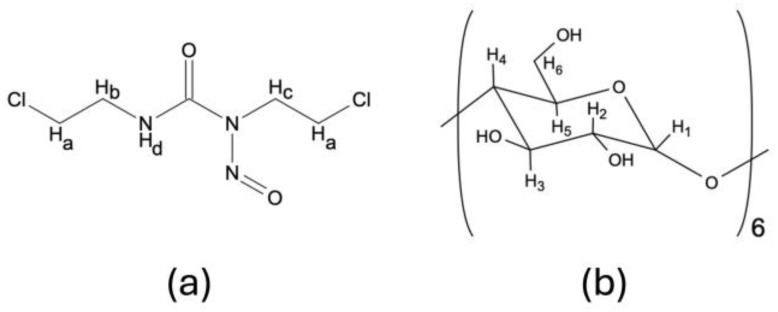
Designation of (**a**) BCNU and (**b**) α-CD protons.

**Figure 3:**
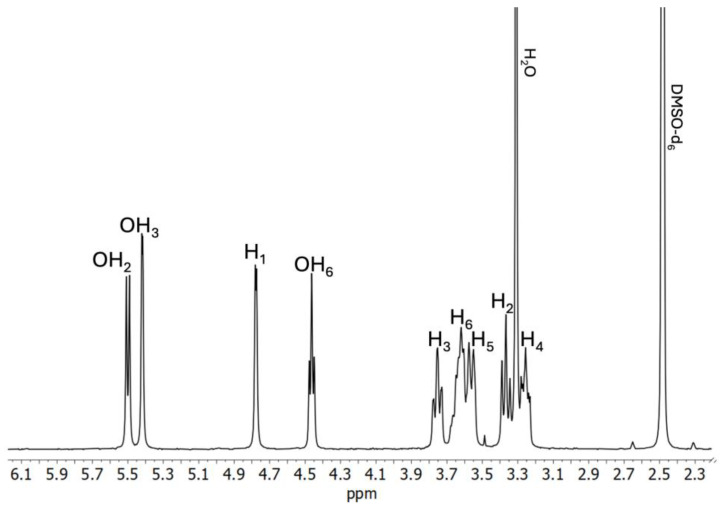
^1^H NMR spectrum of α-CD (DMSO-*d*_6_).

As it was previously described by Lizy Roselet and Prema Kumari, ^1^H NMR may be a valuable tool in the determination of intermolecular interactions between CDs and pharmacologically active substances forming supramolecular complexes [45]. As ^1^H NMR offers the possibility to establish the geometric orientation of the guest molecules in the CD cavity, based on the chemical shift changes (Δδ) of the hydrogen atoms located on the outer surface of CD (H_1_, H_2_, H_4_) and/or hydrogen atoms located within the CD cavity (H_3_, H_5_) (Figure 2), we have performed a thorough analysis of the BCNU-α-CD ICs’ spectra in order to determine Δδ in comparison to the spectra of pure BCNU (Figure S2) and α-CD (Figure 3). The results are presented in Table 2 and Table 3.

The data collected in Table 2 and Table 3 provides information about both the ICs’ formation as well as the depth of the inclusion. The chemical shift changes calculated based on the ^1^H NMR spectra take positive or negative values, which represent a downfield shift or upfield shift, respectively [45]. The most important to consider are Δδ of H_3_ and H_5_. As previously reported, when ΔδH_3_ <ΔδH_5_ a total inclusion occurs, whereas ΔδH_3_ >ΔδH_5_ proves only partial inclusion of the guest molecule [45,46,47].

**Figure 4:**
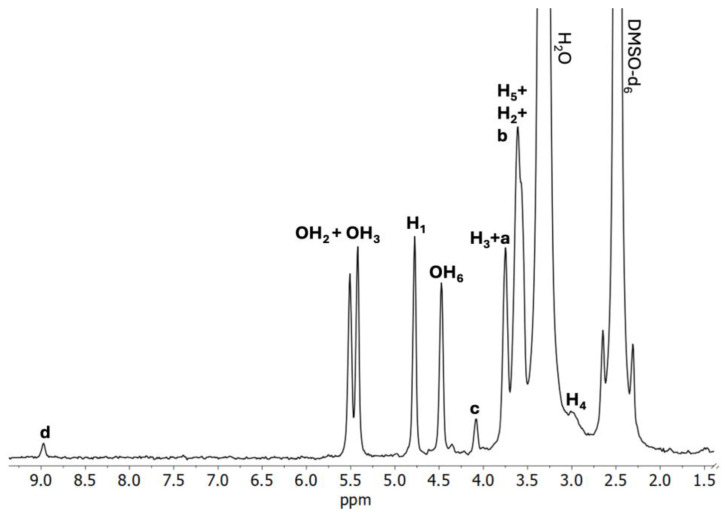
^1^H NMR spectrum of the C1 BCNU-α-CD IC (DMSO-*d*_6_).

When the spectrum of C1 (Figure 4) is considered, a noticeable downfield shift of H_5_ protons occurs, whereas H_4_ protons experienced a remarkable upfield shift. The magnitude of the downfield shift of H_5_ protons, compared to H_3_ protons is slightly greater, which may lead to the conclusion that IC was formed successfully. Considering that the H_b_ protons of BCNU have experienced the upfield shift, while H_a_ and H_d_ are deshielded, it may be assumed that only partial inclusion of BCNU takes place in the α-CD cavity.

**Figure 5:**
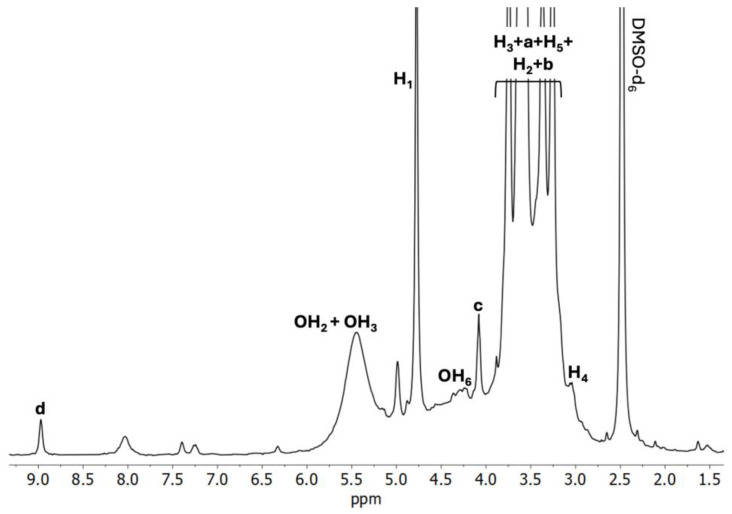
^1^H NMR spectrum of the C2 BCNU-α-CD IC (DMSO-*d*_6_).

Analyzing the C2 spectrum (Figure 5) in relation to the α-CD spectrum reveals that the H_3_ protons exhibit an upfield shift, whereas the H_5_ protons show a downfield shift. In contrast, when comparing the C2 spectrum with the BCNU spectrum, an upfield shift is noted for the H_a_, H_b_, and H_c_ protons from BCNU, while the H_d_ proton displays a downfield shift. These observations further confirm the effective formation of IC and suggest that complete inclusion has occurred.

**Figure 6:**
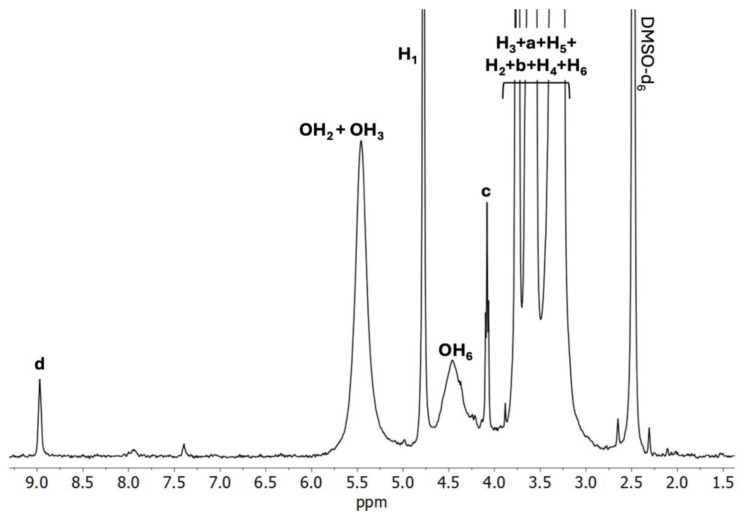
^1^H NMR spectrum of the C3 BCNU-α-CD IC (DMSO-*d*_6_).

Similarly, the C3 IC spectrum (Figure 6) yields results akin to those of the C2 spectrum, with H_3_ protons again showing an upfield shift and H_5_ protons a downfield shift. The upfield shifts observed in the BCNU H_a_ and H_c_ protons reinforce the conclusion of complete inclusion.

#### 2.2.3. ^13^C solid-State NMR Analysis

To further determine the structure of the prepared ICs the ^13^C solid-state NMR was applied. The analysis was performed using two different methods for measuring ^13^C spectra in the solid state. The first sequence (CP) used polarization transfer from hydrogen nuclei to ^13^C carbon nuclei, resulting in the sensitivity gain for ^13^C signals and a decrease in the relaxation time. However, for the effectiveness of the polarization transfer, the Hartman-Hanh condition must be met, which is significantly influenced by the dynamics of analyzed molecule. For highly dynamic molecules the polarization transfer is ineffective, and no signal is observed. This happens for liquid and semi-liquid samples, which is why the CP is effective only for solid phase samples. The second sequence (HPDec—high-power decoupling) is a simple pulse program that does not use the polarization transfer. HPDec, however, lacks sensitivity and requires much more time between the scans for the sample to relax. Nevertheless, HPDec allows for the signal observation regardless of the state of matter.

**Figure 7:**
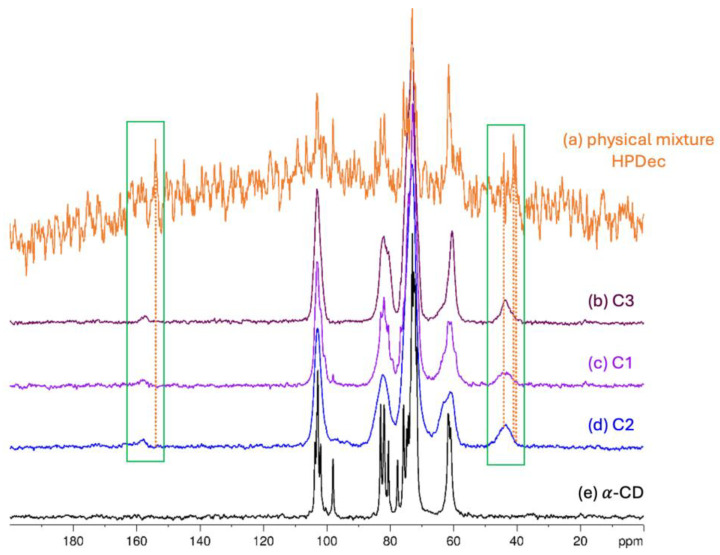
Comparison of ^13^C MAS NMR spectra: (**a**) physical mixture of α-CD and BCNU (1:1) recorded with the HPDec sequence, (**b**) C3, (**c**) C1, (**d**) C2, (**e**) α-CD recorded with the CP sequence.

In the spectrum recorded using the CP sequence for a physical mixture of α-CD and BCNU (Figure 7a and Figure S3), the signals can be solely attributed to the α-CD. The occurrence of this phenomenon may be linked to the temperature within the rotor, which is in the range of 35–40 °C, a consequence of the fast sample spinning (10 kHz) at the magic angle. As the melting point of BCNU is approximately 31 °C, the polarization transfer is simply ineffective. Nevertheless, the HPDec spectra, despite the lower quality, revealed the BCNU presence. The signals in the range of 150–160 ppm and 40–50 ppm can be attributed to the BCNU molecule. For samples C1, C2, C3, however, the CP sequence is effective for both α-CD and BCNU signals, probably because the complex formation influences thermal stability of the BCNU so that it remains in a solid phase under the magic angle spinning conditions.

**Figure 8:**
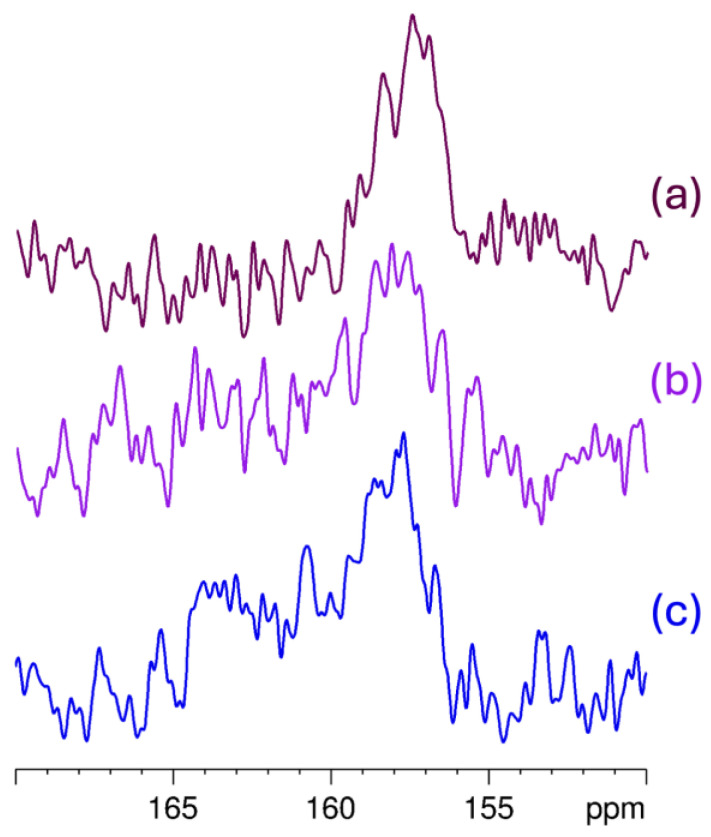
Expansion of the domain of interest in the HPDec CP MAS ^13^C NMR spectra of: (**a**) C3, (**b**) C1 and (**c**) C2.

In Figure 7, the BCNU signals are highlighted by the green frame, which also illustrates their shifts, as indicated by the orange dotted lines, when compared to the spectrum of the physical mixture. The signals exhibit a change in width, which is noticeable in the CP spectra. Additionally, the α-CD signals are broadened significantly in comparison to the pure α-CD spectrum. Notably, the signal at 98 ppm is absent in the C2 and C3 inclusion complex spectra. The C3 spectrum displays the highest intensity of BCNU signals, likely due to the most significant inclusion ratio in this IC. Conversely, the C1 spectrum shows remnants of uncomplexed α-CD, with a faint sharp signal still present at 98 ppm. In the C2 spectrum, the BCNU signals appear wider and less intense. To further elucidate the complexation process, Figure 8 presents an expanded view of the IC spectra within the 150–170 ppm range, where the differences in signal-to-noise ratio and the distribution of chemical shifts associated with the C=O group become more apparent. Again, the best signal-to-noise ratio was observed for C3. Similarly, the width of the ^13^C resonance from C=O is the lowest for C3, indicating a stronger preference for a particular complexation manner.

The ^13^C MAS NMR data provided substantial evidence of the stabilizing effect of α-CD on BCNU. The signals attributed to BCNU were considerably narrower in the spectrum of the physical mixture, likely due to the notable mobility of the BCNU molecule and its partial degradation under the measurement conditions. Conversely, these signals became broader and more pronounced in all of the IC’s spectra. This result indicates that BCNU is more stable when encapsulated in the cavity of α-CD, which enhances its protection against degradation. Importantly, these observations are entirely consistent with the DSC results. The thermograms confirmed the stabilizing influence of α-CD on BCNU, resulting in the disappearance of the characteristic melting peak of BCNU at 31.4 °C across all studied ICs, thereby evidencing the absence of free crystalline drug and confirming successful encapsulation within the cyclodextrin cavity. Additionally, changes in the intensity and shifts in α-CD’s dehydration peaks suggest the replacement of water molecules by BCNU, with this phenomenon being most pronounced in the C3 sample. These findings collectively emphasize the crucial role of the preparation method in determining the stability of the BCNU-α-CD ICs.

#### 2.2.4. FTIR

**Figure 9:**
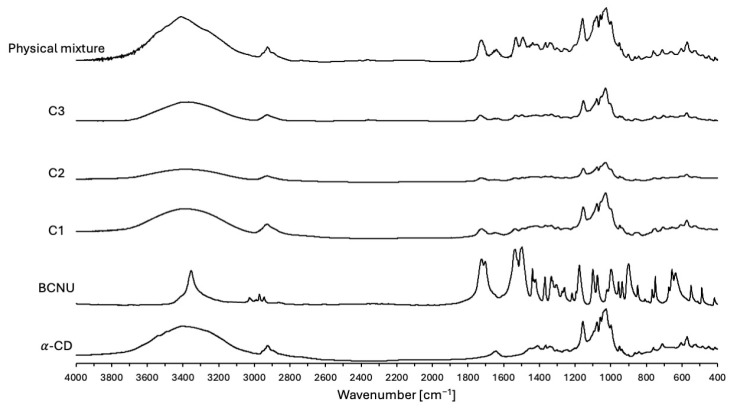
The Fourier transform-infrared (FTIR) spectra of: α-CD, BCNU, physical mixture of α-CD and BCNU in equimolar ratio, C1, C2 and C3.

In order to further substantiate the effective complexation of BCNU, FTIR analysis was undertaken. The FTIR spectra of BCNU, α-CD, physical mixture and BCNU-α-CD ICs prepared by co-grinding (C1), cryomilling (C2) and co-precipitation method (C3) are presented in Figure 9. The comparison between the wavenumbers of pure BCNU, α-CD, prepared physical mixture and BCNU-α-CD ICs is presented in Table S1.

The bands for α-CD observed at 3400 cm^−1^, 2928 cm^−1^ and 1027 cm^−1^ represent the symmetric and anti-symmetric stretching of ν[O-H], ν[C-H] and ν[C-O-C], respectively. The characteristic band at 1157 cm^−1^ depicts the C-O vibrations [48].

The achieved spectrum of BCNU revealed a characteristic band at 1721 cm^−1^ corresponding to the C=O symmetrical stretching vibrations. The bands at 1496 cm^−1^, 1439 cm^−1^ and 1334 cm^−1^ represented the HNC out-of-plane bending, HCH and HCN in-plane bending, respectively. The band at 1176 cm^−1^ was attributed to the HNC out-of-plane bending. Finally, the bands at 997 cm^−1^ and 637 cm^−1^ were assigned to the symmetrical stretching of ν[N-N] and ν[Cl-C], respectively [49]. The FTIR spectra of BCNU-α-CD ICs, as shown in Figure 9, initially appear nearly indistinguishable from the spectrum of pure α-CD. This observation can be explained by the successful formation of the ICs, a phenomenon that has been extensively documented by numerous researchers [50,51]. Additionally, the broad hydroxyl band of pure α-CD at 3400 cm^−1^ was found to be slightly shifted and significantly narrowed in the FTIR spectra of BCNU-α-CD ICs, providing further evidence for the successful establishment of host-guest interactions [50,51,52].

As it has been presented in Table S1, a slight difference, including both increase and decrease in wavenumbers, between the native α-CD, BCNU and BCNU-α-CD ICs has been observed. Special attention needs to be drawn towards the increment in the wavenumber of ν[Cl-C] symmetrical stretching of the BCNU molecule. This phenomenon may arise from an insertion into the electron-dense cavity of α-CD, leading to an increase in the electron cloud density, which in turn results in a rise in frequency [53]. On the other hand, the decrease in the wavenumbers between the ICs and their constituent molecules may appear as a result of hydrogen bonding formation and the occurrence of van der Waals forces, which significantly impact the molecular microenvironment [51].

In the analysis of the FT-IR spectrum for the physical mixture, no notable shifts were detected. This spectrum may be considered as a simple superimposition of BCNU and α-CD bands.

After reviewing all the FTIR data, it can be concluded that the BCNU-α-CD ICs were successfully obtained.

#### 2.2.5. UV-Vis Spectroscopy

The obtained BCNU-α-CD ICs were also investigated by the means of UV-Vis spectroscopy. The complexes were dissolved in the H_2_O:ethanol (50:50) mixture and the absorbance of the prepared solutions was measured in the range of 200–380 nm. Achieved absorption spectra of ICs were then compared with the spectrum of pure BCNU. The wavelengths of maximum absorbance (λ_max_) are presented in Table S2.

The λ_max_ of pure BCNU was equal to 230 nm, which is consistent with the literature data [54]. The λ_max_ of the formulated ICs’ were slightly lower than observed in the pure BCNU sample and revealed the occurrence of hypsochromic shift. Furthermore, a slight increase in the absorbance values for C1 and C3 BCNU-α-CD ICs were observed, while the C2 sample experienced a decrease (Figure S4). As documented in earlier studies, the variations observed in λ_max_ and absorbance values may be attributed to the effective formation of ICs between the drug molecule and the α-CD [29].

#### 2.2.6. ESI-MS Analysis

The characterization of BCNU-α-CD ICs also involved the use of ESI-MS, a technique that is routinely applied to detect noncovalent complex formations [55,56,57]. The ESI-MS analysis has been executed on pure BCNU, as well as on C1, C2, C3 BCNU-α-CD ICs, and the physical mixture of BCNU and α-CD. Unfortunately, the thermal instability of BCNU has led to a limited detection of ions with the expected mass-to-charge ratios. This limitation is likely due to the decomposition of BCNU occurring in the solutions, a consequence of the thermal instability of its molecules. The decomposition mechanisms of BCNU have been thoroughly examined in computational research by Faria et al. and Montgomery et al. [14,58].

In the analysis of the pure BCNU spectrum (Figure S5), we have successfully detected five peaks, which are likely the result of BCNU’s thermal decomposition. The ions at *m/z* 80, 149, 185, 211, and 221 correspond to protonated 2-chloroethanol, potassiated 2-chloroethyldiazene hydroxide, protonated de-nitroso-BCNU, sodiated de-nitroso-BCNU, and a protonated adduct of two molecules of 2-chloroethyldiazene hydroxide. The presence of protonated de-nitroso-BCNU in ESI-MS spectra has also been noted by Kate et al. [59].

The utilization of ESI-MS has yielded insights into the characterization of BCNU-α-CD ICs, uncovering several critical products resulting from thermal decomposition. The detected peaks at *m/z* 149, 211, 995, 1100, and 1967 are likely associated with potassiated 2-chloroethyldiazene hydroxide, protonated de-nitroso-BCNU, sodiated α-CD, the sodiated adduct of α-CD with 2-chloroethyldiazene hydroxide, and the sodiated adduct of two α-CD molecules, respectively. The C1 and C2 spectra exhibited peaks at *m/z* 1208, which may indicate a sodiated adduct of BCNU-α-CD ICs. Additionally, the C3 spectra revealed peaks at *m/z* 215, 1078, 1162, and 2132, potentially corresponding to protonated BCNU, the sodiated adduct of α-CD with 2-chloroethanol, the protonated adduct of α-CD with de-nitroso-BCNU, and a protonated adduct of two α-CD molecules with de-nitroso-BCNU. In contrast, the spectrum of the physical mixture (Figures S6d and S7d) detected protonated 2-ethyldiazene hydroxide, sodiated de-nitroso-BCNU, sodiated α-CD, potassiated α-CD, the protonated adduct of α-CD with 2-chloroethanol, the sodiated adduct of α-CD with de-nitroso-BCNU, and a sodiated adduct of two α-CD molecules at *m/z* values of 149, 211, 995, 1053, 1181, and 1967, respectively [60,61]. These results suggest that the identified products are plausible outcomes of the thermal decomposition of BCNU in the presence of α-CD. Although the findings are encouraging, further investigation using additional analytical methods is advisable to validate these results and gain a comprehensive understanding of the stability and interactions within these complexes.

### 2.3. Determination of Stoichiometry, Stability and Aqueous Solubility

Although the BCNU-α-CD ICs analyzed in our study were sourced in the solid state, we additionally carried out experiments in aqueous solutions to quantitatively evaluate the parameters related to stoichiometry, formation and dissociation constants, solubility enhancement, and drug release profiles. These solution-based studies provide essential insights for predicting the behavior of ICs in biological contexts, thus enhancing the solid-state structural analyses.

#### 2.3.1. Method of Continuous Variation (Job’s Plot)

**Figure 10:**
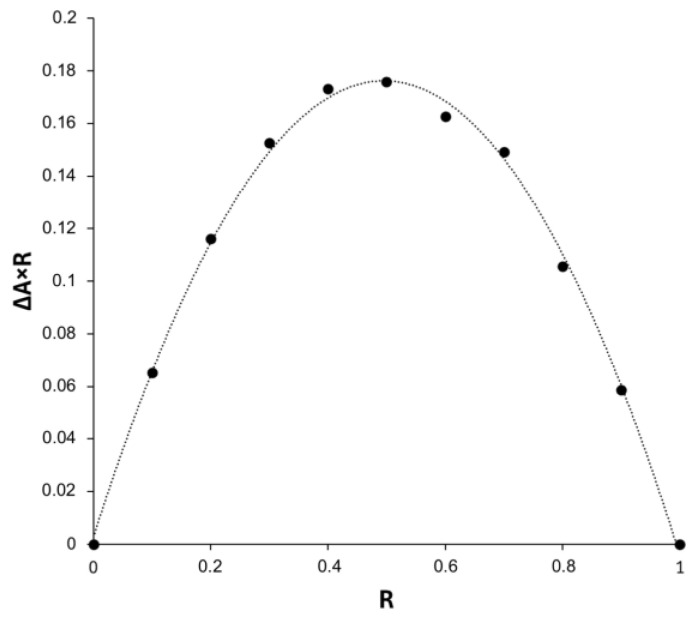
Job’s plot of the BCNU-α-CD IC ([BCNU] + [α-CD] = 0.187 mmol L^−1^) in water solution at 298 K. R = [BCNU]/([BCNU] + [α-CD]), ∆A = the difference in absorbance of BCNU without and with α-CD.

The Job’s method was used to provide the data on the stoichiometry of the BCNU-α-CD ICs. This particular method has been chosen as it is widely used in the determination of various reaction stoichiometries. Figure 10 represents a Job’s plot for the BCNU-α-CD IC examined by the means of UV-Vis spectroscopy.

As it can be seen in Figure 10, the plot has reached maximum at a molar fraction of 0.5, indicating formation of a complex between BCNU and α-CD of 1:1 stoichiometry.

#### 2.3.2. Benesi-Hildebrand Method

To determine the stability of the BCNU-α-CD IC, the Benesi-Hildebrand method was employed. As it can be seen in Figure S8, the plot has an excellent fit to the Benesi-Hildebrand equation, which enables the determination of the dissociation constant (*K*_D_) and the formation constant (*K*_F_). The calculated *K*_D_ and *K*_F_ were equal to 1.35 × 10^−5^ M and 7.55 × 10^4^ M^−1^, respectively.

The high value of calculated *K*_F_ indicates that the interactions occurring between the BCNU and α-CD are relatively strong, exceeding the typically observed values of 10–10^3^ M^−1^ [62]. This remarkable value indicates an exceptional structural compatibility between the BCNU and α-CD. In the study performed by Ma et al., the scientists compared the influence of hydroxypropyl-β-cyclodextrin (HP-β-CD) and sulfobuthylether-β-cyclodextrin (SBE_7_-β-CD) on the stability and solubility of BCNU. The acquired *K*_F_’s from the stability study were equal to 6.3 and 84.0 M^−1^, respectively [13]. The results obtained by Ma and colleagues are lower compared to the value provided by our team. The differences may be acknowledged to the types of CDs used. As reported by Sadaquat and Akhtar in the study focusing on complexation of docetaxel with HP-β-CD, SBE_7_-β-CD and native β-CD, the stability of formed ICs is indeed highly affected by the type of CD used, with the SBE_7_-β-CD exhibiting the highest stabilizing properties (406.41 M^−1^) and the β-CD providing somewhat weaker stabilization (91 M^−1^) [63].

#### 2.3.3. Aqueous Solubility

The aqueous solubility of BCNU and BCNU-α-CD ICs was found to be 1.78 μg/mL and 5.78 μg/mL for C1, 3.37 μg/mL for C2 and 7.63 μg/mL for C3 complexes. The increase in solubility of BCNU after complexation with α-CD was 6.5-fold for C1, 3.8-fold for C2 and 8.6-fold for C3, compared to the solubility of pure BCNU.

Presented results diverge from the observations made by Ma et al., where the intrinsic solubility of BCNU was found to be significantly higher. In the study, Ma et al. chose citrate buffer of a pH equal to 5.5, which may be a cause of acquiring different results [13]. Furthermore, the reason for the low concentrations observed may be due to the degradation of BCNU occurring in an aqueous media [15,64].

Nevertheless, the obtained results confirm that the formation of BCNU-α-CD ICs indeed increases the solubility of the BCNU. This finding is consistent with expectations based on numerous reported cases of solubility enhancements achieved by the complexation of active substances with CDs [65,66]. The enhancement of solubility is a crucial factor to consider regarding the potential of BCNU-α-CD ICs’ use in the medical field, as it significantly broadens the spectrum of their potential applications. This improvement plays a vital role in the enhancement of bioavailability, thus inevitably influencing the potential therapeutic outcomes [67].

### 2.4. BCNU Release Studies

**Figure 11:**
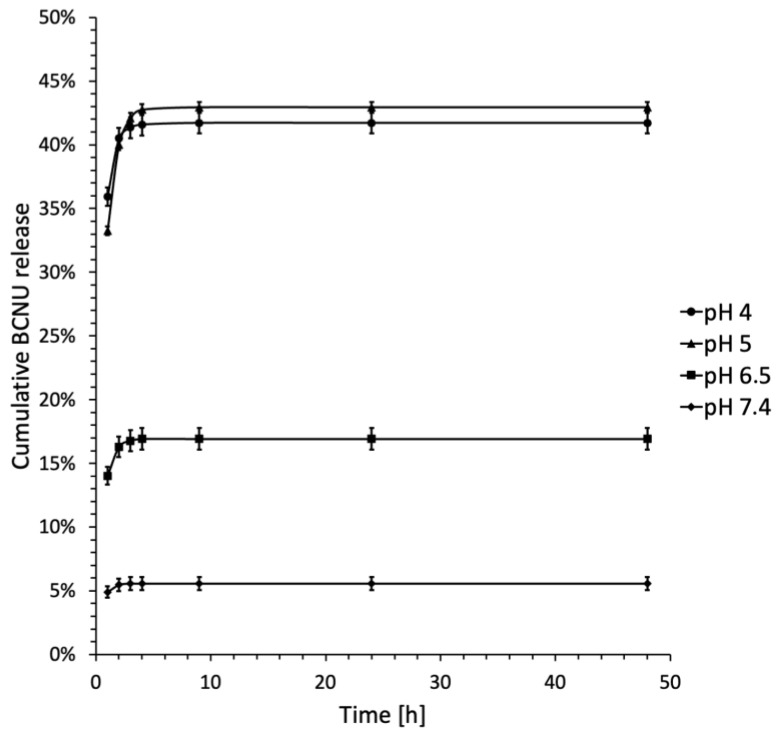
BCNU in vitro release profiles at various pH up to 48 h. Each sample was analyzed in triplicate, and the results are expressed as a mean ± standard error of the mean (SEM).

The evaluation of the therapeutic potential of the developed systems necessitates careful consideration of drug release kinetics. Typically, CD-based formulations do not include a built-in mechanism for regulating drug release, which often results in immediate-release dosage forms [68]. In the context of BCNU, the careful selection of study parameters is vital. Cancer cells are known to have a reversed pH gradient, exhibiting a lower extracellular pH of 6.0 to 7.1 and a higher intracellular pH of 7.4. This necessitates the use of a release media that spans a broad acidic pH range. Moreover, glioma tumors are considerably more acidic, with a pH of approximately 5.9, compared to the normal brain tissue, which maintains a pH of 7.2 to 7.4 [11,69,70,71]. Additionally, the stability of BCNU is closely linked to the pH of the surrounding environment. According to the findings of Laskar and Ayres, BCNU demonstrates optimal stability at pH levels of 5.2 and 5.5 [15]. In our research, we opted to evaluate drug release across a pH spectrum of 4.0 to 7.4. Additionally, since sample C3 demonstrated the highest inclusion ratio and drug loading, aligning with the findings from the ^13^C CP MAS NMR study, it has been selected as the most promising candidate for further investigation. The drug release profiles for sample C3 are depicted as a relationship between cumulative drug release and time (Figure 11).

The data presented in Figure 11 indicates that drug release rates reached a steady state after 4 h across all pH conditions evaluated. At neutral pH, the drug release from the IC was observed to be lower than that at acidic pH, a finding that is consistent with existing literature [11]. The highest release of BCNU was observed at a pH of 5.0, reaching 42.94 ± 0.28%. This was followed by a release of 41.71 ± 0.53% at a pH of 4.0. In contrast, at a pH of 6.5, only 16.92 ± 1.34% of the drug was released, and at the physiological pH of 7.4, the release rate dropped to 5.57 ± 0.78%. These findings align with the research conducted by Athmakur and Kondapi, which also identified the peak BCNU release at a pH of 5.0 [72].

This release profile, which is dependent on pH, is likely influenced by the stability of the BCNU complex and the chemical stability of the drug in different pH environments. Acidic conditions may encourage the dissociation of IC, thus aiding in the release of BCNU, while at physiological pH, the drug’s instability and quick degradation result in a reduced measurable release. This is supported by Loo et al. [73], who found that the half-life of BCNU in plasma was approximately 20 min under in vitro conditions, supporting the idea of rapid drug degradation at neutral pH.

The subsequent phase involved analyzing the outcomes of the BCNU release studies through various models, such as zero-order, first-order, second-order, Higuchi, and Korsmeyer-Peppas models. This analysis aimed to assess the kinetics and mechanisms governing drug release from the synthesized complex. The findings are detailed in Table 4.

The Korsmeyer-Peppas model is widely recognized for determining the release exponent (*n*), which indicates the mechanism of drug release. Specifically, if *n* is less than or equal to 0.45, the drug release is controlled by diffusion (Fickian). When *n* falls between 0.45 and 0.89, the release mechanism is characterized as both diffusion and erosion controlled (anomalous or non-Fickian). Conversely, if *n* is greater than or equal to 0.89, the drug release follows a zero-order or case II transport mechanism [74].

The examination of BCNU release rates from the C3 complex across different pH levels did not produce conclusive results for definitive interpretations. The similar *R*^2^ values observed for zero-order, first-order, and second-order models suggest that no single model is superior in characterizing the release of BCNU from the C3 complex. Notably, all models exhibited the highest *R*^2^ values at pH 7.4, indicating that drug release is the most effective at this pH, irrespective of the model employed. According to the Higuchi model, the *R*^2^ value at pH 7.4 (0.912) implies that diffusion is the predominant mechanism under these conditions. *R*^2^ values exceeding 0.69 at pH levels of 7.4, 6.5, and 5.0 further support the notion that diffusion is the primary mechanism governing drug release in these scenarios. Conversely, the Korsmeyer-Peppas model also recorded the highest *R*^2^ value at pH 7.4 (0.939), suggesting a Fickian release mechanism, as indicated by *n* values ranging from 0.06 to 0.14. This finding reinforces the idea that diffusion is the principal process for BCNU release, which is characteristic of systems where drug release is diffusion controlled [75]. In conclusion, *R*^2^ values exceeding 0.68 in the Korsmeyer-Peppas model and 0.69 in the Higuchi model indicate a strong correlation between experimental data and theoretical models, thereby affirming the validity of these models in elucidating the BCNU release dynamics from the prepared IC.

In conclusion, it is essential to emphasize that our research results align with existing scientific literature. A significant illustration is the investigation of curcumin release from β-cyclodextrin (β-CD) complex, which followed a Fickian diffusion pattern [76]. Additionally, the release of pomalidomide, an anticancer agent used in treating multiple myeloma and Kaposi’s sarcoma, occurred from ICs with β-CD and HP-β-CD, also demonstrating a Fickian diffusion mechanism [77].

### 2.5. Molecular Modeling Studies

#### 2.5.1. Molecular Docking and DFT Calculations

**Figure 12:**
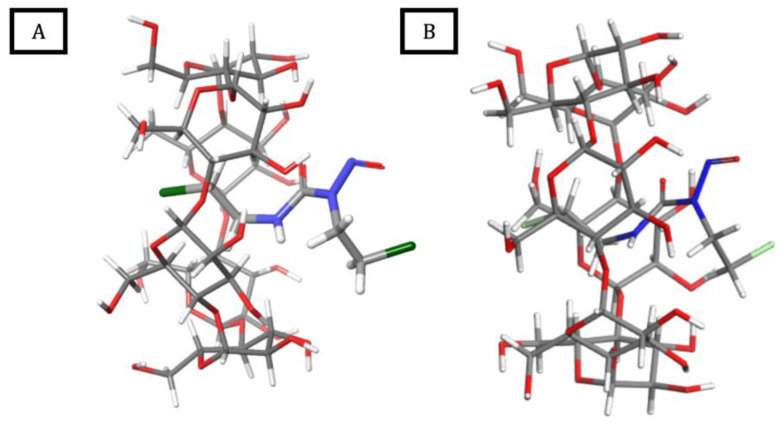
Structures of the complexes formed between α-CD and BCNU from molecular docking (**A**, left) and optimized at the DFT level (**B**, right). Carbon: grey; Hydrogen: white; Oxygen: red; Nitrogen: blue; Chlorine: green.

Figure 12 and Table 5 display the findings from the molecular docking study of BCNU with α-CD, as well as the results obtained from the DFT calculations.

As depicted in Figure 12, the BCNU molecule is ideally accommodated within the α-CD cavity, oriented perpendicularly to the cyclodextrin ring, with its nitroso group protruding through the wider rim of α-CD. The stabilizing effects of electrostatic and van der Waals interactions are substantiated by the negative Glide Score and ΔE values. Thermochemical evaluations performed at the DFT level reveal that the formation of BCNU-α-CD ICs is predominantly an enthalpy-driven process. Although a decrease in entropy is observed during the complexation, this is counterbalanced by a reduction in enthalpy due to the formation of intermolecular interactions. Consequently, the Gibbs free energy for complex formation is negative, indicating that the encapsulation occurs spontaneously.

Additionally, a strong similarity has been noted between the structures of the complexes derived from molecular docking and those optimized at the DFT level, confirming that the geometry of the modeled system is situated at a minimum on the potential energy surface (PES).

Furthermore, the observed proton chemical shift changes (Δδ) for BCNU molecule in the ^1^H NMR studies of BCNU-α-CD ICs are consistent with the molecular orientation of the BCNU within the α-CD’s cavity as predicted by the molecular docking and DFT calculations. The protons of the chloroethyl substituent exhibited the Δδ towards a shielding effect, what has been further confirmed as it is located inside the hydrophobic cavity of α-CD. In contrast, the nitrosourea protons of BCNU displayed only slight Δδ values, suggesting that this part of the molecule remains closer to the rim of α-CD and is more exposed. Thus, the ^1^H NMR data are in complete agreement with the computationally derived orientation of BCNU within the BCNU-α-CD IC. The results of molecular docking and DFT calculations further confirm the possibility of adjustment, reinforcing the consistency between experimental and theoretical findings.

#### 2.5.2. Molecular Dynamics Simulations

**Figure 13:**
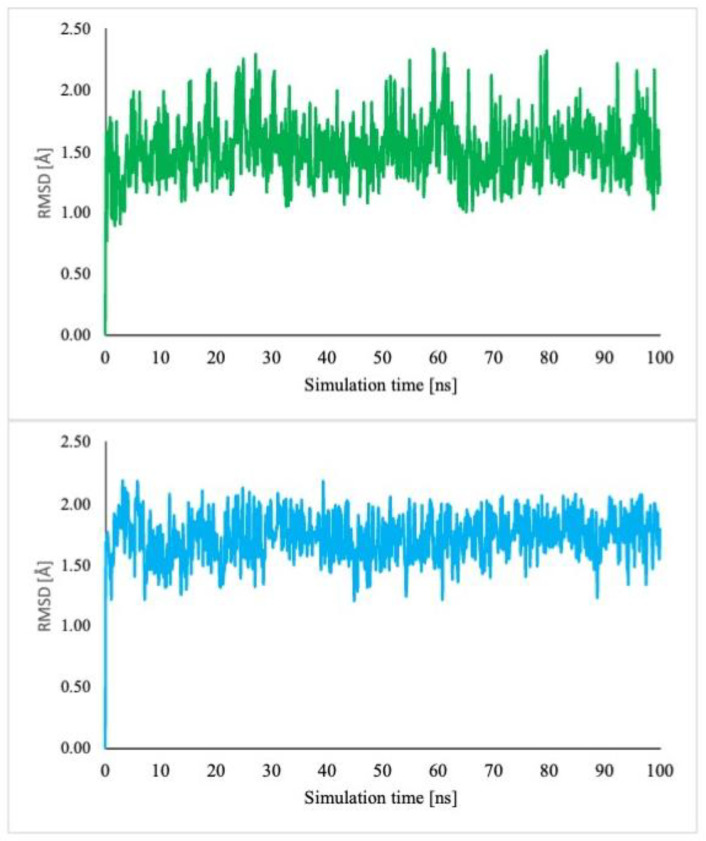
RMSD plot of the host (green) and guest (blue) molecules obtained during the 100 ns MD simulations.

The examination of docking scores and thermodynamic data related to complexation reveals a strong affinity of BCNU for α-CD. MD simulations were conducted to evaluate the dynamic stability of this complex. The BCNU-α-CD ICs were immersed in explicit solvent water, and the simulations were performed at a temperature of 300 K over a duration of 100 ns. To confirm the stability of this inclusion complex, the root mean square deviation (RMSD) was computed relative to the initial frame (Figure 13). The RMSD serves as a metric for assessing complex stability, with a value below 2.0 Å indicating the establishment of a stable system.

The analysis of the frames captured during the simulation period (Video S1) revealed that the guest molecule BCNU exhibits rotational and translational movement along the 7-fold axis of the molecular α-CD. Additionally, conformational alterations of the guest molecule were noted (Figures S9–S11), suggesting that its entropy does not significantly decrease in comparison to non-complexed BCNU. This observation aligns with the findings from Density Functional Theory (DFT), as presented in Table 5. Notably, despite its considerable mobility, the BCNU molecule remained confined within the α-CD cavity throughout the simulation duration. The conclusions drawn were further substantiated by Molecular Mechanics/Generalized Born surface area (MM-GBSA) analysis, with the estimated host–guest binding affinity detailed in Table 5.

## 3. Materials and Methods

### 3.1. Materials

α-Cyclodextrin (α-CD, ≥98% purity), catalog number #PA-03-6443-C was purchased form POL-AURA (Olsztyn, Poland). Carmustine (BCNU, ≥98% purity), catalog number #C2634 was obtained from TCI CO., LTD. (Tokyo, Japan). Trifluoroacetic acid (TFA, 99%), catalog number #T6508 was supplied by Merck (Poznan, Poland). Dimethyl-*d*_6_-sulfoxide (DMSO-*d*_6_) in ampoules, for NMR measurements (99.9 atom% D), was purchased from ARMAR Chemicals (Döttingen, Switzerland). Ethanol, acetonitrile (ACN, HPLC grade min. 99.9%), phosphate buffer solutions ((pH 7.40 ± 0.05, 0.1 M, pH 6.50 ± 0.05, 0.1 M, pH 5.00 ± 0.05, 0.1 M, pH 4.00 ± 0.05, 0.01 M) PBS, and potassium dihydrogen phosphate/di-sodium hydrogen phosphate) were supplied by Avantor (Gliwice, Poland). All the chemicals were used as received.

### 3.2. Methods

#### 3.2.1. Preparation of BCNU-α-CD ICs

##### Co-Grinding

Equal molar quantities of α-CD and BCNU were precisely weighed and combined in a porcelain mortar. A few drops of a 1:1 mixture of ethanol and water were added, and the reagents were mixed thoroughly for 45 min. The solid product obtained was then vacuum dried for 4 days.

##### Cryomilling

A cryogenic impact mill (6770 freezer/mill SPEX CertiPrep, Inc., Metuchen, NJ, USA) was employed for the cryomilling process. Accurately measured quantities of α-CD and BCNU, in equimolar ratios, were added to a vessel containing a stainless-steel rod and immersed in liquid nitrogen. A precooling period of 10 min was applied before initiating the cryomilling. The milling was programmed for 7 cycles, each comprising 6 min of grinding and 3 min of cooling, with an impact frequency set at 15 cycles per second. Once milling was completed, the vial was allowed to equilibrate to room temperature. The resultant solid was then transferred and subjected to vacuum drying for 4 days.

##### Co-Precipitation Method

A precisely measured quantity of α-CD was introduced into a flask, dissolved in water, and stirred on a magnetic stirrer at a stable temperature of 28 °C to create a saturated solution. Subsequently, an ethanolic solution of accurately weighed BCNU, in an equimolar ratio, was added gradually to the saturated α-CD solution, with stirring persisting for 2 h. Following this, the solution was allowed to cool to room temperature and was kept overnight at 4 °C. Ultimately, residual solvents were removed by rotary evaporation, and the precipitated complex was dried under vacuum for 4 days.

The complexation scheme of BCNU within the α-CD cavity using three employed synthesis methods for complexes C1-C3 is presented in the Graphical Abstract.

#### 3.2.2. BCNU Quantification

##### Determination of Inclusion Ratio and Drug Loading (DL)

Analysis was performed on Varian Inc. ProStar^®^ 210 (Harbor City, CA, USA) chromatograph, which included a pump type 210/215/218SD-1, autosampler model 410 (Varian Inc., Harbor City, CA, USA), 325 LC detector (Varian Inc., Harbor City, CA, USA) and Shimadzu (Kyoto, Japan) CTO-10AS VP column oven. Phenomenex (Torrance, CA, USA) C-18 4 mm 3 mm precolumn and Phenomenex (Torrance, CA, USA) Luna C-18 25 cm 4.6 mm (particle size 5 m) column were used in the analysis. The mobile phase consisted of H_2_O:ACN (in ratio of 1:1) with the addition of 0.1% TFA and was used in isocratic mode. The sample injection volume was 40 μL. The analysis was carried out at an auto-sampler and column at a temperature of 25 °C, and flow rate of 1.0 mL/min. The effluent was monitored on the UV detector at a wavelength of 230 nm. BCNU has shown a retention time of about 6 min under these conditions. The calibration curve for BCNU was prepared in a concentration range of 0.25–100 μg/mL (*R*^2^ = 0.9999; y = 81.5343x + 0.8126). To determine the inclusion ratio and drug loading (*DL*), 5.00 mg of inclusion complexes were dissolved in 10 mL of ACN and placed in an ultrasonic bath for about 15 min. Then, the prepared solutions were filtered through 0.22 μm syringe filter. The amounts of BCNU were measured by HPLC, as mentioned above. Inclusion ratio and *DL* were calculated using the following equations:

(1) $$Inclusion\;ratio=\frac{BCNU\;amount\;determined\;by\;HPLC\;[\mu g]}{Total\;amount\;of\;BCNU\;added\;initially\;[\mu g]}\times 100\%$$

(2) $$DL=\frac{BCNU\;amount\;in\;the\;inclusion\;complex\;determined\;by\;HPLC\;[\mu g]}{Total\;amount\;of\;inclusion\;complex\;[\mu g]}\times 100\%$$

##### Solubility Study

The solubility study of BCNU-α-CD ICs was performed in comparison to pure BCNU in distilled water at 25 °C, as described by Arya and Raghaw [48]. A substantial quantity of ICs and pure BCNU was separately introduced into 10 mL glass vials, each containing 2.00 mL of freshly distilled water, and stirred for a duration of 24 h. The vials during the study were covered to protect the samples from the light. Subsequently, the resulting saturated solutions were subjected to centrifugation, and the BCNU concentration was quantified using the HPLC method previously outlined.

##### Method of Continuous Variation (Job’s Plot)

The Job’s method was employed to determine the stoichiometry of the host-guest IC. Briefly, accurately weighted amounts of BCNU and α-CD were dissolved in distilled water, resulting in equimolar concentrations. Then, a set of solutions was prepared, varying the mole fraction (R) of the guest in the range 0–1 (Table S3). The final concentration of each solution remained constant and was equal to 0.187 mmol L^−1^. The absorption spectra were obtained using a UV-Vis spectrophotometer at a temperature of 298 K, with each sample being analyzed in triplicate. The Job’s plot was generated by plotting ∆A × R against the R, where ∆A is the difference in the absorbance between the BCNU solutions with and without α-CD measured at $\lambda_{max}$ = 230 nm, whereas R = [BCNU]/([BCNU] + [α-CD]. The point where the derivative of the curve was equal to zero corresponded to the stoichiometric ratio of guest and host.

##### Benesi-Hildebrand Method

The stability of IC was determined using Benesi-Hildebrand method. Generally, the stability of complexes can be expressed in the matter of its dissociation (*K*_D_) or formation (*K*_F_ = 1/*K*_D_) constants:

(3) $$K_{D}=\frac{\left[S\right][C]}{\left[CS\right]}$$

where [S] represents the concentration of the guest molecule, [C] represents the cyclodextrin concentration and [CS] is the concentration of the inclusion complex.

The *K*_D_ constant of complex can be calculated according to the Benesi-Hildebrand equation [78,79]:

(4) $$\frac{{\left[C\right]}_{0}{\left[S\right]}_{0}}{\Delta A}=\frac{K_{D}}{∆\varepsilon }+\frac{{\left[C\right]}_{0}}{∆\varepsilon }$$

where the absorbance measured (A) is A = A_CS_ + A_C_ + A_S_, so as ∆A = A − ε_C_[C]_0_ − ε_S_[S]_0_ = (ε_CS_ − ε_C_ − ε_S_)*x* = ∆ε*x*. The x is the fraction converted into the inclusion complex.

Therefore, the accurately weighted amounts of BCNU and α-CD were dissolved in distilled water, resulting in concentrations of 2.000 × 10^−4^ M and 1.334 × 10^−3^ M, respectively. In the next step, a set of solutions was prepared, varying the concentration of α-CD, while keeping the BCNU concentration constant (Table S4). The UV-Vis absorption spectra were recorded at 298 K at $\lambda_{max}$ = 230 nm. Each sample was measured in triplicate. By plotting the values of [α-CD]_0_[BCNU]_0_/∆A against [α-CD]_0_ (Figure S8), the value of *K*_D_ was calculated, as it is represented as a quotient between the values of the intercept and the slope [78].

##### In Vitro Release Study of BCNU form BCNU-α-CD ICs

Briefly, approximately 15.00 mg of synthesized BCNU-α-CD IC (C3) was placed in a glass vial and suspended in 1.0 mL of PBS buffer at varying pH levels (7.40 ± 0.05, 6.50 ± 0.05, 5.00 ± 0.05, and 4.00 ± 0.05). The resulting suspension was then transferred into a dialysis membrane (MWCO 2000) and submerged in 10.0 mL of a PBS buffer solution. The release process was conducted at a temperature of 37 °C with a rotational speed of 100 rpm, protecting the samples from the light exposure. At specified time intervals, the release medium was entirely removed for subsequent analysis and replaced with 10.0 mL of fresh, preheated buffer. The collected samples were stored at 2 °C prior to further testing. The BCNU release study was performed over a period of 48 h. Following this, the samples were filtered into glass autosampler vials, sealed, and placed in the HPLC autosampler. The quantity of BCNU released was quantified through HPLC analysis, following the same methodology as used for determining the inclusion ratio, drug loading (*DL*), and solubility. Each sample underwent three tests, and the results were reported as the amount of BCNU released (in μg).

Mathematical models

The BCNU release data points were analyzed using zero-order, first-order, and second-order kinetics, as well as the Higuchi and Korsmeyer–Peppas models. The calculations were performed according to the formulas provided below:

Zero-order:

(5) F=kt

First-order:

(6) $$logF=logF_{0}-\frac{kt}{2.303}$$

Second-order:

(7) $$1/(F_{0}-F)=1/F_{0}-kt$$

Higuchi model:

(8) $$F=k\sqrt{t}$$

Korsmeyer-Peppas model:

(9) $$F=kt^{n}(F<0.6)$$

where

*F* is the fraction of drug released from the matrix after time *t*;
*F*_0_ is the initial amount of drug;
*k* is a model constant and *n* is drug release exponent in Korsmeyer-Peppas model [80,81,82].

### 3.3. Measurements

#### 3.3.1. Structural Examination

All ^1^H NMR measurements were performed using a 700 MHz Agilent DirectDrive2 spectrometer (Santa Clara, CA, USA) equipped with a room-temperature HCN probe, temperature-controlled at 25 °C. For the suppression of water signal, PRESAT pulse sequence with a number of transients = 16, interscan delay = 2 s, acquisition time = 2.94 s, saturation power = −15.5 dB, saturation delay = 2 s and 32 K complex points was used. Samples of BCNU-α-CD ICs were dissolved in DMSO-*d*_6_.

**Data for ^1^H NMR of**α**-CD** (DMSO-*d*_6_, 400 MHz, δH, ppm): 5.51–5.49 (OH_2_), 5.42 (OH_3_), 4.78–4.77 (H_1_), 4.46 (OH_6_), 3.75 (H_3_), 3.62 (H_6_), 3.58–3.55 (H_5_), 3.37 (H_2_), 3.26 (H_4_).

**Data for ^1^H NMR of BCNU** (DMSO-*d*_6_, 400 MHz, δH, ppm): 8.97 (-N**H**-, **d**), 4.08 (-CH_2_-C**H_2_**-N-, **c**), 3.75 (Cl-C**H_2_**-, **a**), 3.61 (-CH_2_-C**H_2_**-NH-, **b**).

**Data for ^1^H NMR of C1** (DMSO-*d*_6_, 400 MHz, δH, ppm): 8.99 (-N**H**- of BCNU, **d**), 5.51 (OH_2_ of α-CD), 5.42 (OH_3_ of α-CD), 4.77 (H_1_ of α-CD), 4.48 (OH_6_ of α-CD), 4.08 (-CH_2_-C**H_2_**-N- of BCNU, **c**), 3.77 (Cl-C**H_2_**- of BCNU, **a**), 3.75 (H_3_ of α-CD), 3.61 (-CH_2_-C**H_2_**-NH- of BCNU, **b** and H_6_ of α-CD), 3.57 (H_5_ of α-CD), 3.40 (H_2_ of α-CD), 3.01 (H_4_ of α-CD).

**Data for ^1^H NMR of C2** (DMSO-*d*_6_, 400 MHz, δH, ppm): 8.98 (-N**H**- of BCNU, **d**), 5.46 (OH_2_ and OH_3_ of α-CD), 4.77 (H_1_ of α-CD), 4.56 (OH_6_ of α-CD), 4.07 (-CH_2_-C**H_2_**-N- of BCNU, **c**), 3.74 (Cl-C**H_2_**- of BCNU, **a** and H_3_ of α-CD), 3.60 (-CH_2_-C**H_2_**-NH- of BCNU, **b** and H_6_ of α-CD), 3.58 (H_5_ of α-CD), 3.39 (H_2_ of α-CD), 3.27 (H_4_ of α-CD).

**Data for ^1^H NMR of C3** (DMSO-*d*_6_, 400 MHz, δH, ppm): 8.97 (-N**H**- of BCNU, **d**), 5.46 (OH_2_ and OH_3_ of α-CD), 4.78 (H_1_ of α-CD), 4.46 (OH_6_ of α-CD), 4.07 (-CH_2_-C**H_2_**-N- of BCNU, **c**), 3.74 (Cl-C**H_2_**- of BCNU, **a** and H_3_ of α-CD), 3.66 (H_6_ of α-CD), 3.61 (-CH_2_-C**H_2_**-NH- of BCNU, **b**), 3.58 (H_5_ of α-CD), 3.38 (H_2_ of α-CD), 3.24 (H_4_ of α-CD).

#### 3.3.2. UV-Vis

UV-Vis spectra were recorded utilizing a UV 1202 Shimadzu (Kyoto, Japan) spectrophotometer. The samples of BCNU and BCNU-α-CD ICs were prepared in a 50:50 mixture of H_2_O and ethanol and subsequently placed in 1.00 cm quartz cells. The spectral data were collected over a wavelength range of 200 to 380 nm.

#### 3.3.3. FTIR

The FTIR spectra of BCNU, α-CD, their physical mixture (1:1 molar ratio), and BCNU-α-CD ICs were recorded using the KBr pellet technique within the range of 400 to 4000 cm^−1^. The scanning conditions were set to 25 scans, with a resolution of 2.0 cm^−1^ and an interval of 1.0 cm^−1^.

#### 3.3.4. DSC

The thermal properties of the produced materials were analyzed via DSC. For this purpose, a Q200 calorimeter manufactured by TA Instruments (New Castle, DE, USA) was utilized. The samples of BCNU-α-CD ICs and physical mixture underwent heating from 0 °C to 230 °C at a heating rate of 10 °C/min. The BCNU analysis was carried out at a heating rate of 5 °C/min in a range from 0 °C to 70 °C. The analysis was carried out at a nitrogen flow of 25 mL/min.

#### 3.3.5. ESI-MS

High-resolution mass spectrometry (HRMS) analyses were conducted utilizing the Synapt G2-Si mass spectrometer (Waters, Milford, MA, USA), which is outfitted with an ESI source and a quadrupole-time-of-flight mass analyzer. The mass spectrometer functioned in the positive ion detection mode. The parameters for the optimized source included a capillary voltage of 3.6 kV, a cone voltage of 60 V, a source temperature of 120 °C, a desolvation gas (nitrogen) flow rate of 750 L/h at a temperature of 450 °C, and a nebulizer gas pressure of 6.5 bar. All samples were dissolved in a water-methanol solution (1:1) and introduced into the instrument via a standard electrospray ion source. The scanning range was set to *m/z* 50–3000, with an acquisition method runtime of 1 min. To guarantee precise mass measurements, data were gathered in centroid mode, and mass was adjusted during acquisition using a leucine enkephalin solution as an external reference (Lock-Spray^TM^, Waters, Milford, MA, USA), which produced a reference ion at *m/z* 556.2771 Da ([M + H]^+^) in positive ESI mode. The measurement results were analyzed using the MassLynx 4.1 software (Waters) integrated with the instrument.

#### 3.3.6. ^13^C CP MAS NMR

The ^13^C CP MAS NMR and ^13^C high-power decoupling MAS NMR experiments were conducted on solid samples of α-CD, BCNU-α-CD ICs, and a physical mixture of α-CD and BCNU using a Bruker (Billerica, MA, USA) Avance III 400 spectrometer. This instrument operates at resonant frequencies of 100.63 MHz for ^13^C and 400.19 MHz for ^1^H. The samples were contained in 4 mm zirconia rotors and spun at a speed of 10 kHz under the magic angle. A Bruker 4 mm wide-bore probe head was utilized for all measurements, with spectra referenced to the resonance of adamantane, set at δ = 38.48 ppm. The relaxation delay was established at 2 s for the CP MAS experiments and 60 s for the high-power decoupling experiment. The latter was particularly useful for analyzing the physical mixture, allowing for the detection of resonances from non-complexed BCNU, which may not be efficiently observed under MAS conditions due to cross-polarization limitations.

#### 3.3.7. Molecular Modeling Studies

##### Initial Structures Preparation

Coordinates of α-CD and BCNU were extracted from their crystal structures, downloaded from CCDC [https://www.ccdc.cam.ac.uk/structures, accessed on 24 January 2025], refcodes: RIRZUN and QENWOZ01, respectively.

##### Molecular Docking

1. Grid generation

To facilitate grid generation, the mass center of α-CD served as the focal point of the grid. A cubic box was constructed with dimensions adequate to completely contain the ligand. The grid for α-CD was produced using the standard parameters for the van der Waals scaling factor (1.00) and charge cutoff (0.25) within the OPLS4 force field framework.

2. Ligand preparation

For the docking process, the ligand BCNU was prepared utilizing LigPrep from the Schrödinger Maestro 12.8 Suite, with the OPLS4 force field applied for geometry optimization. The remaining settings in LigPrep were left at their default configurations.

3. Glide XP docking

Flexible docking was performed using the Grid-based Ligand Docking with Energetics (Glide) module from the Schrӧdinger Maestro 12.8. Suite, using the OPLS4 forcefield and extra precision (XP) scheme. Glide score (GScore) was calculated as GScore = 0.05 × vdW + 0.15 × Coul + Lipo + Hbond + Metal + Rewards + RotB + Site, wherein vdW: van der Waals energy; Coul: Coulomb energy; Lipo: Lipophilic term; Hbond: Hydrogen-bonding term; Metal: Metal-binding term; Rewards: Rewards and penalties for various features, such as buried polar groups, hydrophobic enclosure, correlated hydrogen bonds, amide twists, and so on.; RotB: Penalty for rotatable bonds that have been frozen; Site: active site polar interactions.

##### Molecular Dynamics Simulations

Molecular dynamics (MD) simulations were performed using Desmond program of Schrӧdinger Maestro package version 12.8. (Schrӧdinger, LLC, New York, NY, USA, 2023). The host-guest complex with the optimal Glide score was chosen as the basis for MD simulations. The Desmond System Builder module was employed for this purpose. The BCNU-α-CD IC was positioned in an orthorhombic box, and the systems were solvated with water using the TIP3P model, ensuring a buffer distance of 10 Å. Following this, the system was subjected to the steepest descent minimization in accordance with Desmond’s default protocol prior to commencing the MD simulations.

The relaxation protocol comprises eight sequential stages, starting with the minimization of solute heavy atoms under restraints, followed by a phase of minimization without restraints. This is followed by a simulation that gradually heats the system from 0 K to 300 K, the implementation of a water barrier with a gradual restraining process, and an NPT equilibration simulation that includes a water barrier while restraining heavy atoms. Subsequently, the solvent undergoes NPT equilibration, and the protocol continues with a simulation under the NPT ensemble where the restraint on host heavy atoms is reduced from 10.0 to 2.0 kcal/mol. The final stage involves a 1.5 ns simulation under the NPT ensemble with no restraints applied.

Following the relaxation phase, unrestrained simulations were conducted for a duration of 100 ns. These simulations were executed within the NPT ensemble framework, utilizing the Nose-Hoover thermostat to ensure a stable temperature of 300 K, alongside the isotropic Martyna-Tobias-Klein barostat to maintain a pressure of 1 atm. Short-range Coulombic interactions were assessed with a cut-off distance of 9.0 Å, employing a short-range method. A time-reversible reference system propagator algorithm (RESPA) integrator was applied with a time step of 2.0 fs, and trajectory data was recorded at 100 ps intervals for subsequent analysis. Upon completion of the simulations, RMSD values were calculated using the Simulation Interaction Diagram from the Schrödinger Suite. The binding free energy (dG) was determined using the Molecular Mechanics Generalized Born Surface Area (MM-GBSA) approach, specifically with the Prime VSGB2.0 implicit water model and the OPLS4 force field. The thermal_mmgbsa.py script facilitated the computation of MM-GBSA binding energy for frames extracted from the MD simulation trajectories, with 500 frames selected from the final 50 ns of simulation time. These calculations were performed after removing water molecules and isolating the CAR from the α-CD, as specified in the thermal_mmgbsa.py script.

##### Quantum Chemical Calculations

Geometry optimization of uncomplexed α-CD and BCNU, as well as the complex obtained from MD, has been performed at the density functional theory (DFT) level using the Gaussian 16 software [https://gaussian.com/products/, accessed on 26 January 2025]. All electron calculations were performed using the large 6-311++G(d,p) basis set, B3LYP functional and Grimme D3 empirical dispersion correction. The polarizable continuum model (PCM) was used for implicit solvation, choosing water as the solvent with dielectric constants 78.540 [83]. In our recent work, we have shown that this approach provides accurate results for the calculations of CDs complexes [84].

To ensure that each structure was not in a transition state, the natural mode frequencies were determined through harmonic approximation. The identification of solely positive frequencies validated the presence of stationary points on the potential energy surface. This calculation of vibrational frequencies facilitated the estimation of zero-point vibrational energy (ZPVE) corrections and thermodynamic parameters, such as Gibbs free energy (G), enthalpy (H), and temperature-corrected entropy (TS), at 298.15 K and 101.325 kPa.

To investigate the changes in the analyzed values resulting from complexation, calculations were performed according to the equation ΔA = A(BCNU@α-CD) − [A(BCNU)) + A(α-CD)], where “A” is either E, H, TS or G.

## 4. Conclusions

The development of DDSs has been a key factor in the conversion of innovative therapeutic modalities into effective clinical solutions. As the therapeutic landscape has progressed, DDSs and their foundational technologies have rapidly evolved to meet the increasing complexities of drug administration. The delivery strategies are primarily influenced by the physicochemical characteristics of the drugs, which crucially affect their bioavailability. The application of advanced carriers and nanotechnology-based platforms, enables substantial improvement in the solubility of active substances, thereby enhancing both bioavailability and therapeutic efficacy. Furthermore, encapsulating sensitive compounds within specially designed matrices protects them from degradation and may help mitigate the adverse effects of microenvironmental pH, ensuring more consistent pharmacokinetic profiles and optimized therapeutic outcomes.

Aligned with this objective, our investigation was focused on the preparation and comprehensive characterization of ICs of BCNU with α-CD. Although BCNU exhibits excellent activity as an antineoplastic agent, its administration is hindered by its poor aqueous solubility and pronounced hydrolytic instability, leading to rapid degradation in solution and necessitating stringent storage conditions. Furthermore, BCNU’s short plasma half-life further limits the therapeutic efficacy, as the drug is quickly eliminated from systemic circulation. These challenges significantly complicate the formulation and handling of BCNU, underscoring the need for advanced DDSs to optimize its clinical use. Therefore, in our study, we have successfully obtained BCNU-α-CD ICs in the solid state for the first time utilizing three different synthesis approaches: co-grinding, cryomilling and co-precipitation. The selected synthesis methodologies exerted a pronounced influence on the reaction yield, inclusion ratio and *DL* values. The highest yield was achieved via the co-grinding method, while the highest inclusion ratio and *DL* values were recorded for the co-precipitation approach. To elucidate the precise orientation of the guest molecule BCNU within the α-CD cavity, an in-depth analysis was conducted employing ^1^H NMR spectroscopy. A comparison of the chemical shift values of protons associated with BCNU and α-CD against those observed in the spectra of the ICs revealed that co-grinding resulted in partial inclusion of BCNU. In contrast, both cryomilling and co-precipitation processes enabled a complete encapsulation of BCNU within the cavity of α-CD.

The complexation of BCNU was further corroborated by complementary spectroscopic techniques, including FT-IR, UV-Vis, ^13^C CP MAS NMR and ESI-MS. Furthermore, solubility studies, as well as comprehensive assessments of BCNU-α-CD ICs stoichiometry and stability, were undertaken employing the Job’s method of continuous variation and the Benesi-Hildebrandt approach, respectively. The performed analysis yielded results that the complexation of BCNU within α-CD cavity indeed enhances its solubility and the stoichiometry of BCNU-α-CD ICs was confirmed as 1:1 by Job’s plot. Regardless of the synthesis method employed, BCNU and α-CD form a 1:1 complex in solution. The observed differences between samples pertain only to the solid state, as corroborated by the results from ^1^H NMR, ^13^C CP MAS NMR, DSC and FTIR analyses. The distinctions, revealed by the multimodal spectroscopic and DSC, directly influenced the formulation properties, such as inclusion ratio, *DL*, thermal stability. The most favorable parameters were observed for the co-precipitation method. Therefore, this sample was chosen for further drug release assessment. Moreover, the DSC and solid-state NMR studies demonstrated that α-CD acts as a stabilizer for BCNU, protecting it from degradation and decomposition. Conducted in vitro drug release studies demonstrated that the BCNU was released in a controlled, sustained, pH-dependent manner primarily via Fickian diffusion mechanism. The highest cumulative release was observed in the acidic microenvironment (42.94 ± 0.28% at pH of 5.0), which aligns with the existing literature regarding the stability of the BCNU.

Employing the molecular docking calculations, the structure of BCNU-α-CD IC has been calculated. It was observed that the BCNU is located deeply inside the CD cavity, perpendicular to its ring, with the nitroso group protruding through the wider rim. Geometry optimization at the DFT level confirmed the geometry received from MM calculations and explained the formation of this inclusion complex as being enthalpy driven. The BCNU-α-CD IC remained stable through the long, 100 ns MD simulations in aqueous solution, which indicates the stability of this system. Furthermore, the results obtained from ^1^H NMR analyses, along with molecular docking and DFT calculations, were in total agreement, confirming the molecular orientation of BCNU and its effective encapsulation within the α-CD cavity for the samples generated through cryomilling and co-precipitation.

In conclusion, the BCNU-α-CD ICs present a promising platform for the development of prolonged release formulations of BCNU, which could potentially be utilized in GBM treatment. Among the samples investigated, the IC obtained through the co-precipitation method is particularly notable, as it integrates a high inclusion ratio, stabilization of the labile BCNU molecule, and a controlled release profile, thereby underscoring the pharmaceutical potential of α-CD-based DDSs.

## Figures and Tables

**Table 1:** Synthesis parameters and conditions for the formation of BCNU-α-CD ICs.

Sample	Synthesis Method	Yield [%]	Inclusion Ratio ^1^ [%]	*DL* ^2^ [%]
C1	Co-grinding	96	50.31	2.71
C2	Cryomilling	72	49.27	2.95
C3	Co-precipitation	92	85.65	15.2

The experiments were conducted using equimolar ratios of BCNU and α-CD (1:1). ^1^ Inclusion ratio—the percentage of the BCNU entrapped in the α-CD relative to the initial amount of BCNU used in the synthesis; ^2^ *DL*—drug loading.

**Table 2:** Chemical shifts (δ) of α-CD protons and their difference (Δδ) in the presence of BCNU, depending on the synthesis method.

H	α-CD	BCNU-α-CD IC					
	$\delta_{\alpha -CD}$	$\delta_{\alpha -CD-C1}$	Δδ _C1_	$\delta_{\alpha -CD-C2}$	Δδ _C2_	$\delta_{\alpha -CD-C3}$	Δδ _C3_
H_1_	4.78	4.77	−0.01	4.77	−0.01	4.78	0.00
H_2_	3.37	3.40	+0.03	3.39	+0.02	3.38	+0.01
H_3_	3.75	3.75	0.00	3.74	−0.01	3.74	−0.01
H_4_	3.26	3.01	−0.25	3.27	+0.01	3.24	−0.02
H_5_	3.55	3.57	+0.02	3.58	+0.03	3.58	+0.03
H_6_	3.62	3.61	−0.01	3.60	−0.01	3.66	+0.04
OH_2_	5.50	5.51	+0.01	5.46	−0.04	5.46	−0.04
OH_3_	5.42	5.42	0.00	5.46	+0.04	5.46	+0.04
OH_6_	4.46	4.48	+0.02	4.56	+0.10	4.46	0.00

The spectra were recorded using DMSO-*d*_6_ as a solvent.

**Table 3:** Chemical shifts (δ) of BCNU protons and their difference (Δδ) in presence of α-CD, depending on the synthesis method.

H	BCNU	BCNU-α-CD IC					
	$\delta_{BCNU}$	$\delta_{BCNU-C1}$	Δδ _C1_	$\delta_{BCNU-C2}$	Δδ _C2_	$\delta_{BCNU-C3}$	Δδ _C3_
a	3.75	3.77	+0.02	3.74	−0.01	3.74	−0.01
b	3.61	3.60	−0.01	3.60	−0.01	3.61	0.00
c	4.08	4.08	0.00	4.07	−0.01	4.07	−0.01
d	8.97	8.99	+0.02	8.98	+0.01	8.97	0.00

The spectra were recorded using DMSO-*d*_6_ as a solvent.

**Table 4:** Evaluation of the data pertaining to the release of BCNU from the formulated BCNU-α-CD IC (C3).

No.	pH	Zero-Order Model	First-Order Model	Second-Order Model	Higuchi Model	Korsmeyer-Peppas Model		Release Kinetics	Drug Transport Mechanism
		***R* ^2^**	***R* ^2^**	***R* ^2^**	***R* ^2^**	***R* ^2^**	***n***		
C3	4	0.306	0.325	0.347	0.640	0.685	0.06	*	rather Fickian diffusion
C3	5	0.357	0.387	0.420	0.696	0.731	0.11	*	Fickian diffusion
C3	6.5	0.637	0.648	0.659	0.881	0.899	0.14	*	Fickian diffusion
C3	7.4	0.700	0.703	0.705	0.912	0.939	0.13	*	Fickian diffusion

* Small differences in the calculation results obtained for individual models (zero-, first- or second-order model).

**Table 5:** MM and QC results obtained for the complex formed between BCNU and α-CD. Glide Score—score obtained from molecular docking, MM-GBSA ΔG—Molecular Mechanics Generalized Born Surface Area binding free energy, ΔE—energy of complexation, ΔG—Gibbs free energy of complexation, ΔH—enthalpy of complexation, TΔS—temperature corrected entropy of complexation. All the values are in kcal/mol.

	MM Level		QC Level—DFT			
BCNU-α-CD ICs	Glide Score	MM-GBSA ΔG	ΔE	ΔG	ΔH	TΔS
	−1.833	−38.54	−22.95	−5.74	−20.75	−15.01

## Data Availability

The raw and processed data underlying this article will be shared on reasonable request to the corresponding author.

## Supplementary Materials

The supporting information can be downloaded at: https://www.mdpi.com/article/10.3390/ijms26199386/s1.

## Abbreviations

The following abbreviations are used in this manuscript:

$\lambda_{max}$	Maximum absorption wavelength
^13^C CP MAS NMR	Carbon-13 Cross-Polarization Magic Angle Spinning Nuclear Magnetic Resonance
^1^H NMR	Proton nuclear magnetic resonance
A	Absorbance
ACN	Acetonitrile
BBB	Blood–brain barrier
BCNU	Carmustine
C1	Co-grinding method
C2	Cryomilling method
C3	Co-precipitation method
DDSs	Drug delivery systems
DFT	Density functional theory
DL	Drug loading
DMSO-*d*_6_	Dimethyl-*d*_6_-sulfoxide
DSC	Differential scanning calorimetry
ESI-MS	Electrospray Ionization Mass Spectrometry
*F*	Fraction of drug released from the matrix after time t
*F*_0_	Initial amount of drug
FDA	U.S. Food and Drug Administration
FT-IR	Fourier-transform infrared spectroscopy
G	Gibbs free energy
GBM	Glioblastoma multiforme
H	Enthalpy
HPDec	High-power decoupling
HPLC	High-performance liquid chromatography
HP-β-CD	Hydroxypropyl-β-cyclodextrin
HRMS	High-resolution mass spectrometry
ICs	Inclusion complexes
*k*	Model constant
*K*_D_	Dissociation constant
*K*_F_	Formation constant
MD	Molecular dynamics
MM	Molecular mechanics
MM-GBSA	Molecular Mechanics Generalized Born Surface Area
MM-GBSA ΔG	Molecular Mechanics Generalized Born Surface Area binding free energy
n	Drug release exponent in Korsmeyer-Peppas model
PBS	Phosphate-buffer solution
PCM	Polarizable continuum model
PES	Potential energy surface
QC	Quantum chemical
R	Mole fraction
RMSD	Root mean square deviation
SBE_7_-β-CD	Sulfobuthylether-β-cyclodextrin
TFA	Trifluoroacetic acid
*T*_m_	Melting temperature
TMZ	Temozolomide
TS	Temperature-corrected entropy
TΔS	Temperature corrected entropy of complexation
UV-Vis	Ultraviolet-visible spectroscopy
ZPVE	Zero-point vibrational energy
α-CD	α-cyclodextrin
β-CD	β-cyclodextrin
ΔA	Difference in the absorbance
ΔE	Energy of complexation
ΔG	Gibbs free energy of complexation
ΔH	Enthalpy of complexation
Δδ	Chemical shift change
