# Supplementary material for: Investigation of Host-Guest Inclusion Complexes Between Carmustine and α-Cyclodextrin: Synthesis, Characterization, and Evaluation
Source: Int J Mol Sci. 2025 Sep 25;26(19):9386. doi: 10.3390/ijms26199386 (PMC12524515; doi:10.3390/ijms26199386)
Supplement: Supplementary file 1 [file ijms-26-09386-s001.zip › Supporting Information Oledzka E et al Revised version.pdf]

Supporting information

Investigation of host-guest inclusion complexes between carmustine and α-cyclodextrin: synthesis, characterization, and evaluation

Katarzyna Strzelecka [1,2], Dominika Janiec [1], Jan Sobieraj [1,2], Adam Kasiński [1], Marzena Kuras [1], Aldona Zalewska [3], Łukasz Szeleszczuk [4], Marcin Sobczak [1], Marta K. Dudek [5], Ewa Oledzka [1*]

[1] Department of Pharmaceutical Chemistry and Biomaterials, Faculty of Pharmacy, Medical University of Warsaw, 1 Banacha Street, 02-097 Warsaw, Poland
[2] Doctoral School, Medical University of Warsaw, 81 Żwirki i Wigury Street, 02-093 Warsaw, Poland
[3] Chair of Inorganic Chemistry, Faculty of Chemistry, Warsaw University of Technology, 3 Noakowskiego Street, 00-664 Warsaw, Poland
[4] Department of Organic and Physical Chemistry, Faculty of Pharmacy, Medical University of Warsaw, 1 Banacha Street, 02-097 Warsaw, Poland
[5] Structural Studies Department, Centre of Molecular and Macromolecular Studies, Polish Academy of Sciences, 112 Sienkiewicza Street, 90-363 Łódź, Poland

*Corresponding Author: eoledzka@wum.edu.pl

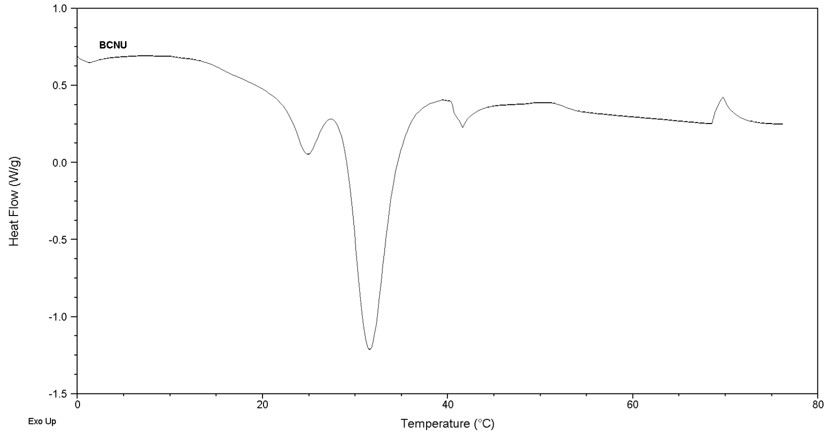

Figure S1. DSC thermogram of BCNU.

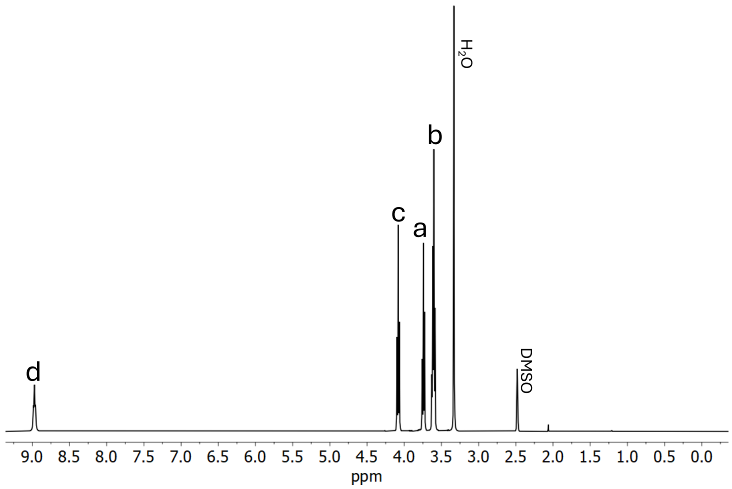

Figure S2. ^{1}H NMR spectrum of BCNU.

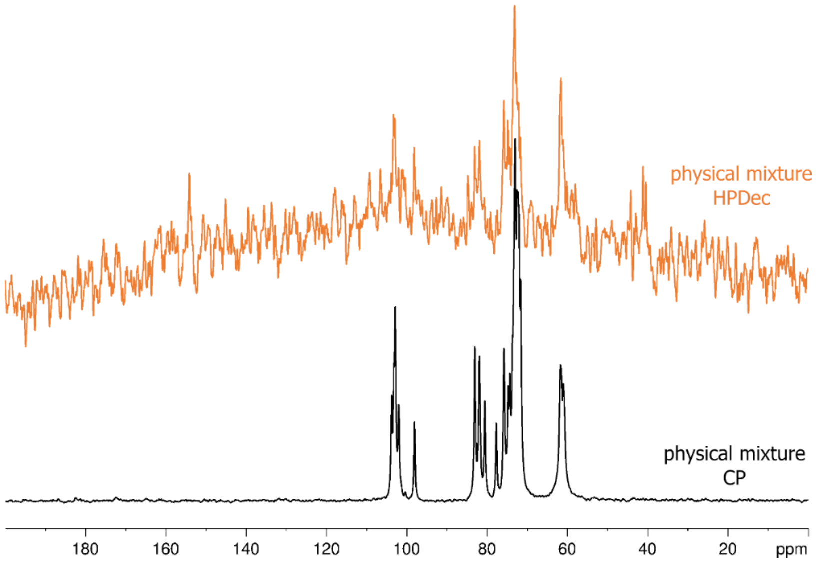

Figure S3. CP MAS ^{13}C NMR spectra of the physical mixture of α-CD and BCNU (1:1) recorded using two different pulse sequences: (a) HPDec and (b) CP.

Table S1. Comparison between the wavenumber of BCNU, α-CD, physical mixture and inclusion complexes.

Functional group	Wavenumber [cm^{-1}]					
	BCNU	α-CD	Physical mixture	C1	C2	C3
ν[O-H] stretching	-	3400	3410	3384	3376	3378
ν[C-H]	-	2928	2928	2925	2926	2927
C-O vibrations	-	1157	1157	1154	1152	1154
ν[C-O-C] stretching	-	1027	1027	1027	1029	1028
ν[C=O] stretching, in-plane bending of CCH	-	952	952	949	950	950
ν[Cl-C] symmetrical stretching	637	-	640	647	658	655
ν[N-N] symmetrical stretching, in-plane bending of CCH	997	-	997	999	1003	1001
HNC out-of-plane bending	1176	-	1157	1154	1152	1154
HCN in-plane bending	1334	-	1334	1332	1333	1331
HCH in-plane bending	1439	-	1439	1448	1420	1420
HNC out-of-plane bending	1496	-	1496	1497	1496	1496
ν[C=O] symmetrical stretching	1721	-	1721	1731	1719	1725

Table S2. UV-Vis wavelengths of maximum absorbance for BCNU and ICs.

Sample	λ_{max} [nm]
BCNU	230
C1	228
C2	229
C3	229

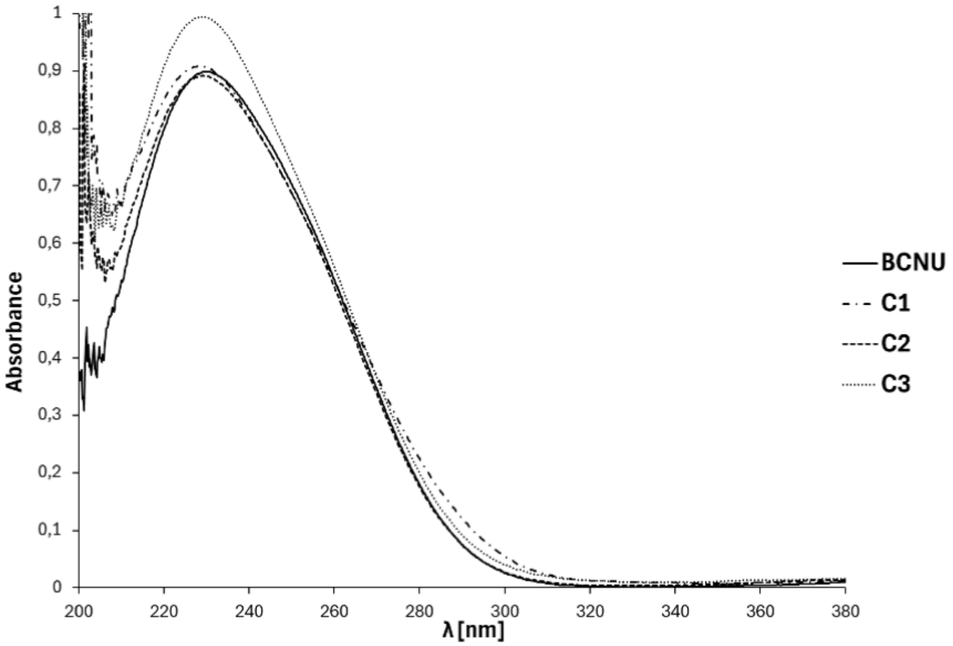

Figure S4. UV-Vis spectra of BCNU, C1, C2 and C3.

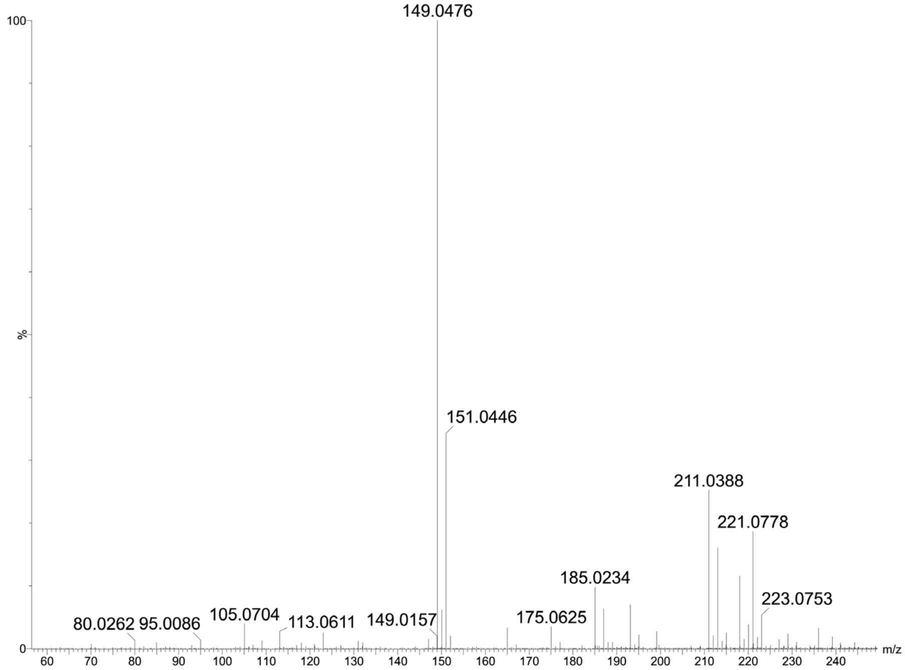

Figure S5. ESI-MS spectrum of pure BCNU.

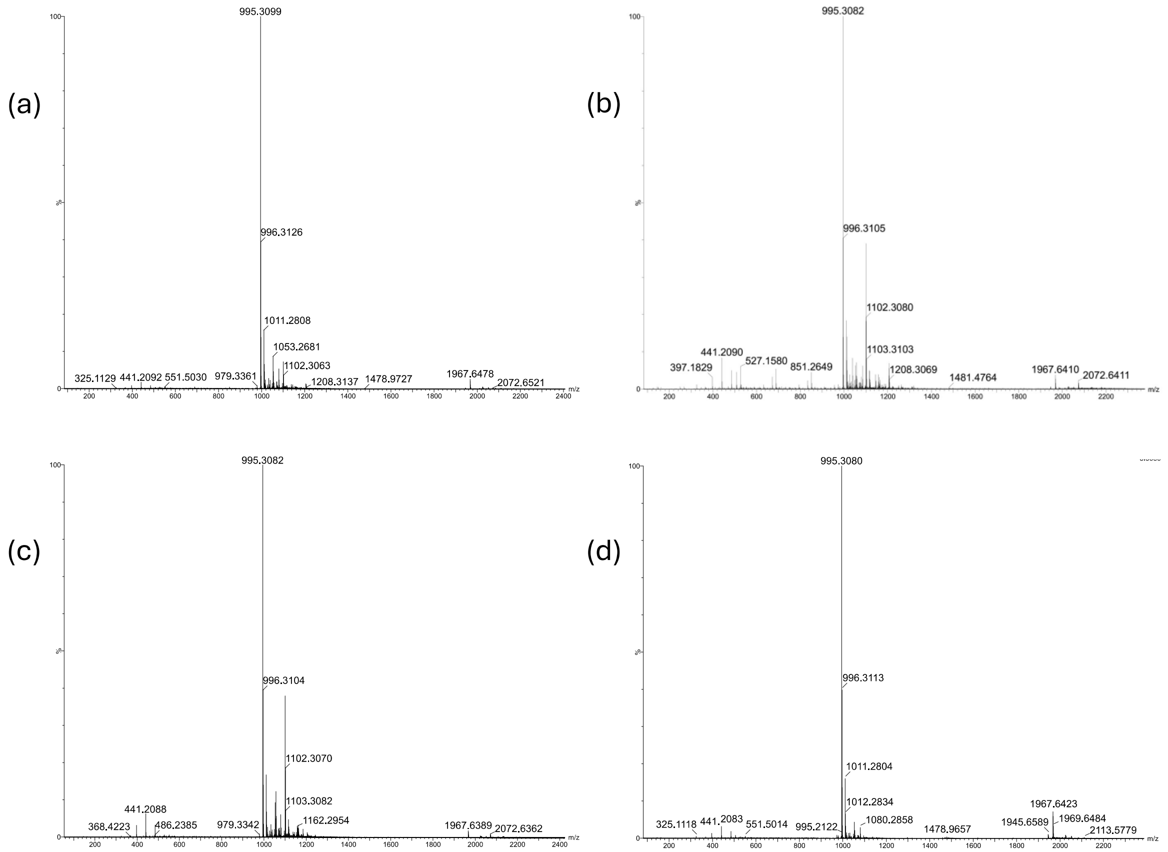

Figure S6. ESI-MS spectra of: (a) C1, (b) C2, (c) C3 and (d) physical mixture.

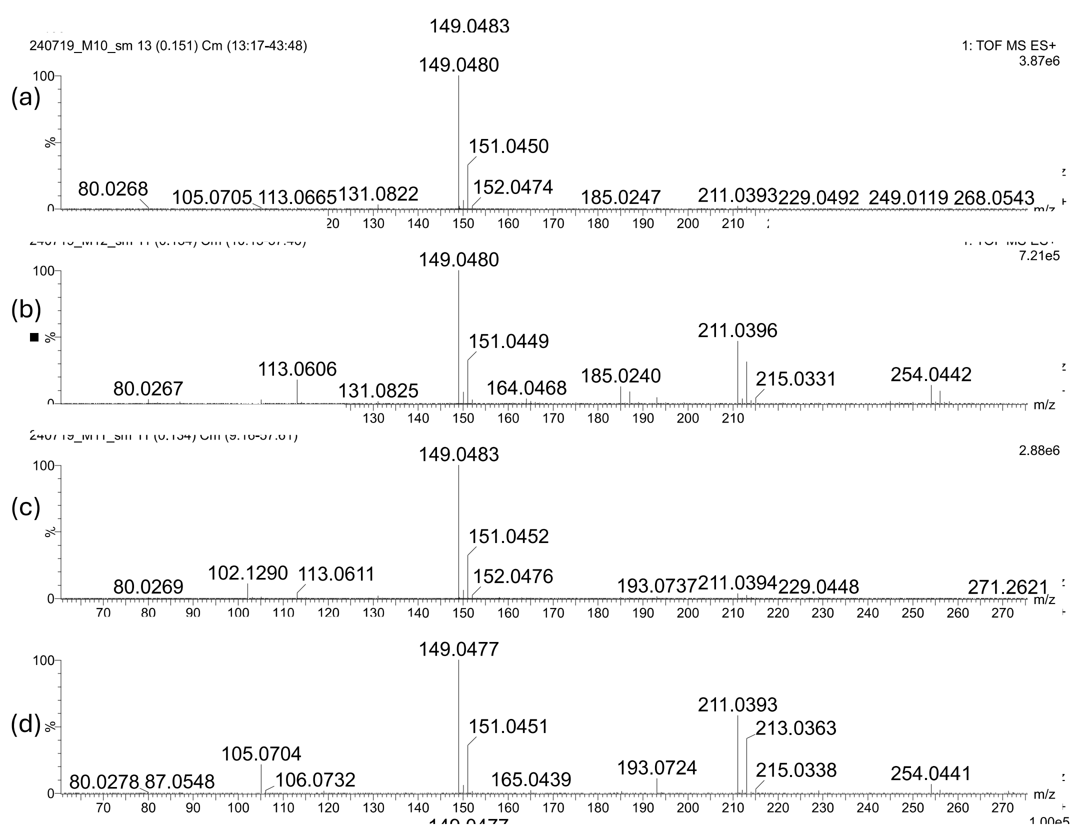

Figure S7. ESI-MS spectra of: (a) C1, (b) C2, (c) C3 and (d) physical mixture in the lower range of *m/z* ratios.

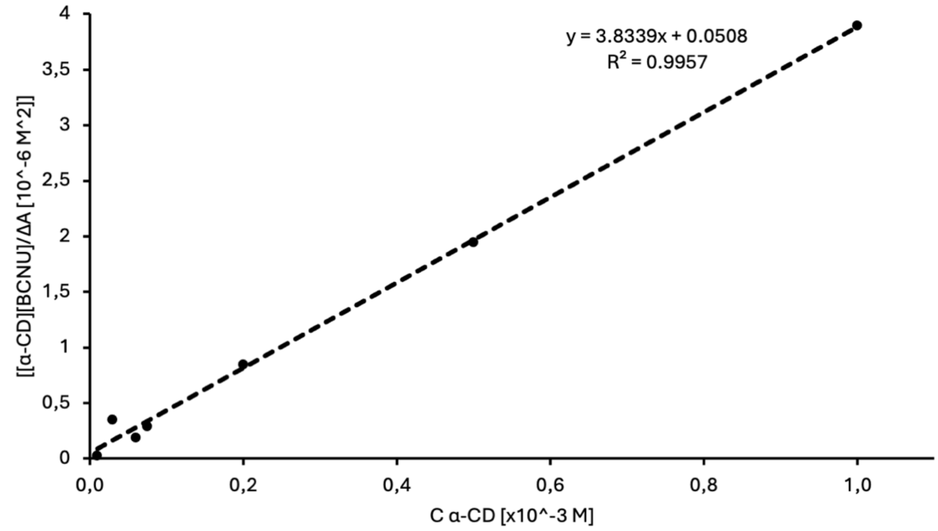

Figure S8. Determination of the equilibrium constant of the BCNU- α-cyclodextrin complex according to the Benesi-Hildebrand equation at 230 nm.

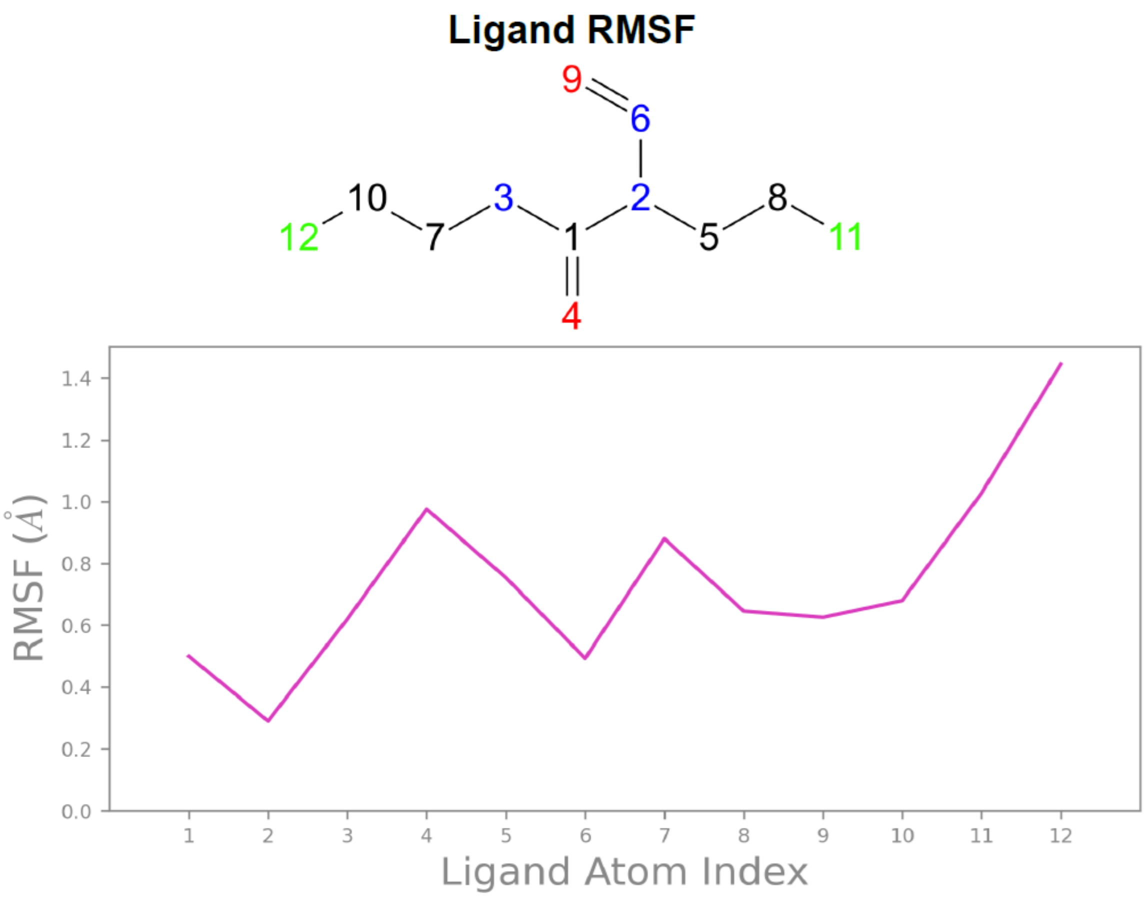

Figure S9. Ligand RMSF. The Ligand Root Mean Square Fluctuation (L-RMSF) is useful for characterizing changes in the ligand atom positions. The RMSF for atom i is:

$$RMSF_i = \sqrt{\frac{1}{T}\sum_{t=1}^{T}(r_i'(t)) - r_i(t_{ref}))^2}$$

where T is the trajectory time over which the RMSF is calculated, t_{ref} is the reference time (first frame, regarded as the zero of time); r is the position of atom i in the reference at time t_{ref}, and r' is the position of atom i at time t after superposition on the reference frame. Ligand RMSF shows the ligand's fluctuations broken down by atom, corresponding to the 2D structure in the top panel. The ligand RMSF provides insights on how ligand fragments interact with the α-CD and their entropic role in the binding event. In this case the chlorine atoms of BCNU were characterized by the highest dynamics, while the inner part of this molecule was quite rigid, as it remains inside the CD cavity during entire simulation.

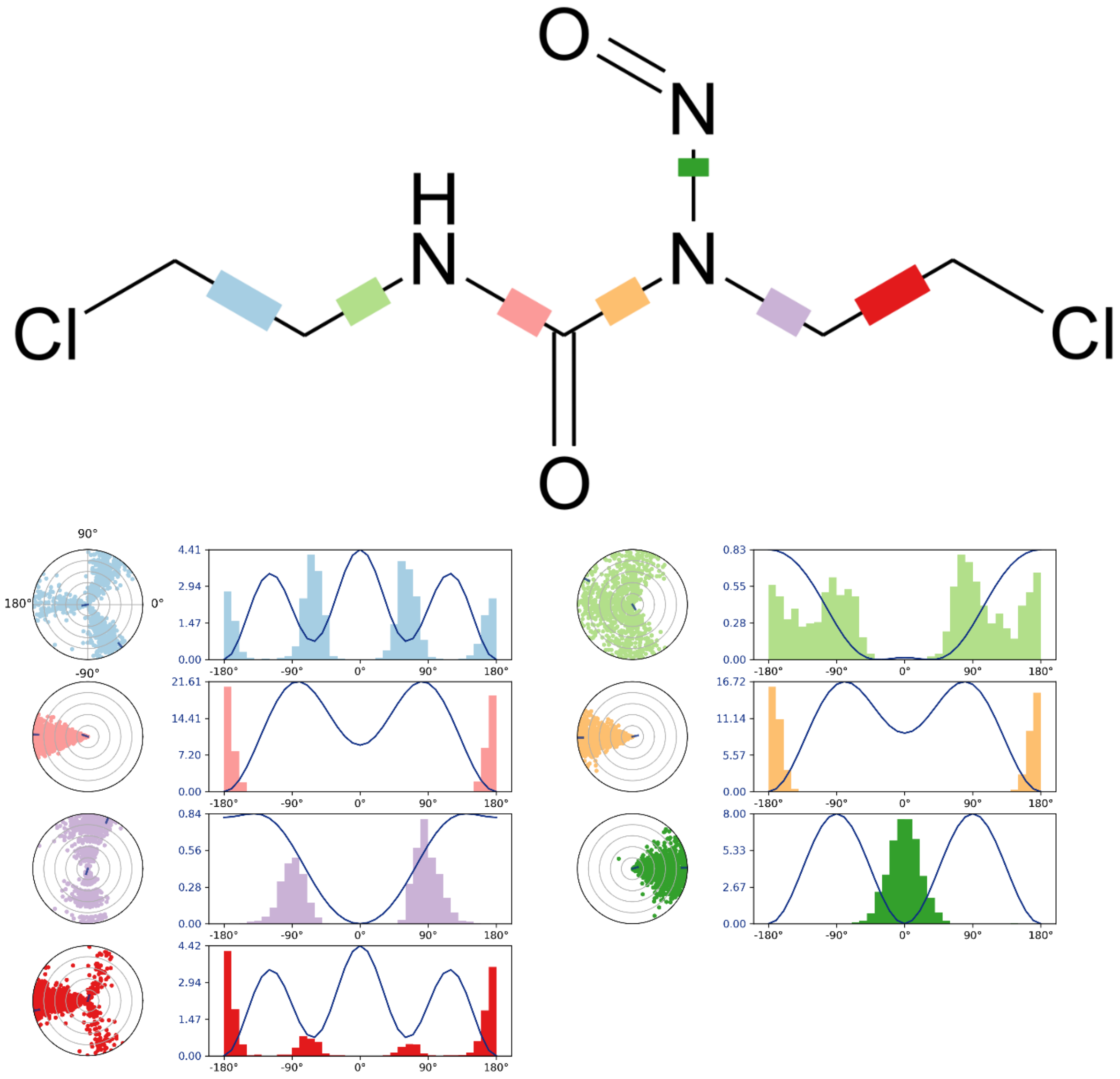

Figure S10. Ligand Torsion Profile. The ligand torsions plot summarizes the conformational evolution of every rotatable bond (RB) in the ligand throughout the simulation trajectory (0.00 through 100.00 nsec). The top panel shows the 2d schematic of a ligand with color-coded rotatable bonds. Each rotatable bond torsion is accompanied by a dial plot and bar plots of the same colour. Dial (or radial) plots describe the conformation of the torsion throughout the course of the simulation. The beginning of the simulation is in the center of the radial plot and the time evolution is plotted radially outwards. The bar plots summarize the data on the dial plots, by showing the probability density of the torsion. If torsional potential information is available, the plot also shows the potential of the rotatable bond (by summing the potential of the related torsions). The values of the potential are on the left Y-axis of the chart, and are expressed in kcal/mol. Looking at the histogram and torsion potential relationships may give insights into the conformational strain the ligand undergoes to maintain a α-CD-bound conformation of BCNU.

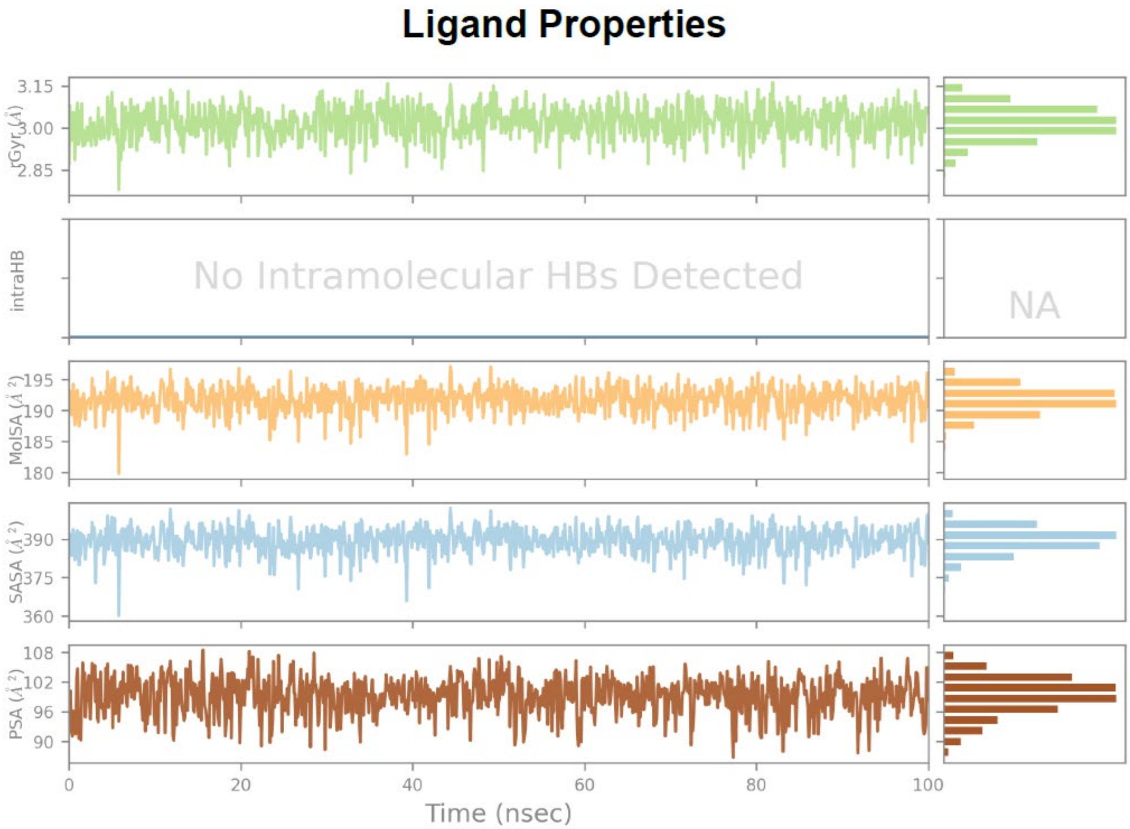

Figure S11. Ligand Torsion Profile. **Radius of Gyration** (rGyr): Measures the 'extendedness' of a ligand, and is equivalent to its principal moment of inertia. **Intramolecular Hydrogen Bonds** (intraHB): Number of internal hydrogen bonds (HB) within a ligand molecule. **Molecular Surface Area** (MolSA): Molecular surface calculation with 1.4 Å probe radius. This value is equivalent to a van der Waals surface area. **Solvent Accessible Surface Area** (SASA): Surface area of a molecule accessible by a water molecule. **Polar Surface Area** (PSA): Solvent accessible surface area in a molecule contributed only by oxygen and nitrogen atoms.

Table S3. Data for the Job's plot performed by UV-Vis spectroscopy for aqueous BCNU-α-CD inclusion complex at 298 K.

**BCNU [μL]**	**α-CD [μL]**	**BCNU [mmol L^{-1}]**	**α-CD [mmol L^{-1}]**	**[BCNU]/([BCNU]+[α-CD] (R)**	**Absorbance (A)**	**ΔA**	**ΔA×R**
0	3000	0	0.187	0.0	0.000	0.743	0.000
300	2700	0.019	0.168	0.1	0.090	0.653	0.065
600	2400	0.038	0.149	0.2	0.162	0.581	0.116
900	2100	0.056	0.131	0.3	0.234	0.509	0.153
1200	1800	0.075	0.112	0.4	0.310	0.433	0.173
1500	1500	0.093	0.094	0.5	0.391	0.352	0.176
1800	1200	0.112	0.075	0.6	0.472	0.271	0.163
2100	900	0.131	0.056	0.7	0.530	0.213	0.149
2400	600	0.149	0.038	0.8	0.611	0.132	0.106
2700	300	0.168	0.019	0.9	0.678	0.065	0.059
3000	0	0.187	0	1.0	0.743	0.000	0.000

Table S4. Data for the Benesi-Hildebrand method performed by UV-Vis spectroscopy for aqueous BCNU-α-CD inclusion complex at 298 K.

**BCNU [μL]**	**α-CD [μL]**	**BCNU [$\cdot 10^{-3}$ M]**	**α-CD [$\cdot 10^{-3}$ M]**	**H_2O [μL]**	**Absorbance (A)**	**ΔA**	**[α-CD]$_0$[BCNU]$_0$/ΔA**	**Slope**	**Intercept**
750	22.5	0.05	0.01	2227.5	0.297	0.018	0.02595	3.8339	0.0508
	67.5		0.03	2182.5	0.283	0.004	0.35029		
	135.0		0.06	2115.0	0.294	0.015	0.18682		
	168.8		0.08	2081.2	0.291	0.012	0.29200		
	450.0		0.2	1800.0	0.290	0.011	0.84919		
	1125.0		0.5	1125.0	0.291	0.012	1.94607		
	2250.0		1.0	0	0.291	0.012	3.89213		
	0		0	2250.0	0.279	-	-		
